# Suspension cell cultures of *Panax vietnamensis* as a biotechnological source of ginsenosides: growth, cytology, and ginsenoside profile assessment

**DOI:** 10.3389/fpls.2024.1349494

**Published:** 2024-02-26

**Authors:** Maria V. Titova, Maria K. Lunkova, Tatiana M. Tyurina, Olga N. Prudnikova, Elena V. Popova, Oleg I. Klychnikov, Pavel S. Metalnikov, Yuri A. Ikhalaynen, Elizaveta N. Vasileva, Igor A. Rodin, Alexander M. Nosov

**Affiliations:** ^1^ K.A. Timiryazev Institute of Plant Physiology, Russian Academy of Sciences, Moscow, Russia; ^2^ Department of Biochemistry, Faculty of Biology, M.V. Lomonosov Moscow State University, Moscow, Russia; ^3^ Department of Analytical Chemistry, Faculty of Chemistry, M.V. Lomonosov Moscow State University, Moscow, Russia; ^4^ Department of Plant Physiology, Faculty of Biology, M.V. Lomonosov Moscow State University, Moscow, Russia

**Keywords:** Vietnamese ginseng, plant cell culture, plant biotechnology, ginsenosides, suspension cell culture

## Abstract

**Introduction:**

*Panax vietnamensis* is a valuable medicinal plant and a source of a broad spectrum of biologically active ginsenosides of different structural groups. Overexploitation and low adaptability to planation cultivation have made this species vulnerable to human pressure and prompted the development of cell cultivation *in vitro* as a sustainable alternative to harvesting wild plants for their bioactive components. Despite high interest in biotechnological production, little is known about the main factors affecting cell growth and ginsenoside biosynthesis of this species under *in vitro* conditions. In this study, the potential of cell cultures of *P. vietnamensis* as a biotechnological source of ginsenosides was was assessed.

**Methods:**

Six suspension cell lines that were developed from different sections of a single rhizome through a multi-step culture optimization process and maintained for over 3 years on media with different mineral salt base and varying contents of auxins and cytokinins. These cell lines were evaluated for productivity parameters and cytological characteristics. Ginsenoside profiles were assessed using a combination of the reversed-phase ultra-high-performance liquid chromatography–Orbitrap–tandem mass spectrometry (UHPLC–Orbitrap–MS/MS) and ultra-performance liquid chromatography–time of flight–mass spectrometry (UPLC–TOF–MS).

**Results:**

All lines demonstrated good growth with a specific growth rate of 0.1–0.2 day^−1^, economic coefficient of 0.31–0.70, productivity on dry weight (DW) of 0.30–0.83 gDW (L·day)^−1^, and maximum biomass accumulation varying from 10 to 22 gDW L^−1^. Ginsenosides of the protopanaxadiol (Rb1, Rb2/Rb3, malonyl-Rb1, and malonyl-Rb2/Rb3), oleanolic acid (R0 and chikusetsusaponin IV), and ocotillol (vinaginsenoside R1) groups and their isomers were identified in cell biomass extracts. Chikusetsusaponin IV was identified in *P. vietnamensis* cell culture for the first time.

**Discussion:**

These results suggest that suspension cell cultures of Vietnamese ginseng have a high potential for the biotechnological production of biomass containing ginsenosides, particularly of the oleanolic acid and ocotillol groups.

## 1 Introduction

*Panax vietnamensis* Ha et Grushv., or Vietnamese ginseng, was officially recognized as a species in 1985. The plant is endemic to Vietnam and southern China and is the southernmost species of the genus (Ha and Grushvitzky, 1985; Pham et al., 2019; Liu et al., 2020; Vu et al., 2020). Vietnamese ginseng is a rich source of a specific group of triterpene glycosides called ginsenosides, which are common for *Panax* spp (Nguyen et al., 2020). A wide spectrum of triterpene glycosides reported for *P. vietnamensis* includes ginsenosides Rb1, Rb2, Rb3, Rc, Rd, Re, Rg1, Rh1, Rh4, Rh5, and XVII; majonosides R1, R2, and F1; notoginsenosides Fa, R1, R2, and R6; vinaginsenosides R1–R25; ginsenoside R0; hemsloside Ma3; pseudoginsenosides RS1, RT4, RC1, and F11; 20-gluco-ginsenoside-Rf; 20(R)-ginsenoside-Rh1; hypenosides XVII and IX; quinquenoside R1; and protopanaxatriol oxide sapogenin II (Duong et al., 2013; Xia et al., 2022). In addition to typical ginsenosides of the protopanaxadiol (PPD) and protopanaxatriol (PPT) groups and oleanolic acid derivatives, *P. vietnamensis* contains significant amounts of ocotillol-type ginsenosides (OCTs) (Zhang et al., 2015; Ha et al., 2016). Other bioactive compounds of the plant include polysaccharides, peptides, polyacetylenes, fatty acids, and essential oils (Nguyen et al., 2020; Xia et al., 2022).

Underground parts of *P. vietnamensis* have traditionally been used in Vietnamese folk medicine to improve and restore physical strength and to treat fatigue, hepatitis, diabetes, and intoxication (Banskota et al., 2003; Vu et al., 2020). Recent studies demonstrated that bioactive components of *P. vietnamensis* help regulate blood sugar and treat trachoma and possess hepatoprotective effects (Nguyen et al., 2000; Tran et al., 2002). Ocotillol derivatives of *P. vietnamensis* demonstrated antimelanogenic activity (Xia et al., 2022) and inhibited inflammatory processes such as colitis (Jeong et al., 2015; Lee et al., 2015). The anticancer activity of *P. vietnamensis* also tends to correlate with the accumulation of ocotillol-type ginsenosides (Vo et al., 2015; Nguyen and Phuong, 2019; Xia et al., 2022). The significant amounts of majonoside R2 in *P. vietnamensis* contribute to its anti-stressor, antidepressant, hepatoprotective, and anxiolytic (sedative) effects; stimulation of psychomotor functions; and memory improvement (Dela Peña et al., 2017; Xia et al., 2022). Due to the presence of majonoside R2, extracts of Vietnamese ginseng suppressed stress-induced antinociception and exerted protective effects against stress-induced gastric lesions (Nguyen and Phuong, 2019). Vinaginsenosides R2 and R7 demonstrated therapeutic effects on neuroinflammation (Xia et al., 2022).

The qualitative and quantitative compositions of ginsenosides in wild *P. vietnamensis* vary significantly among different plant parts (Van Le et al., 2015). For example, majonoside R2, the major saponin in roots, was not detected in leaf extracts (Van Le et al., 2015). Underground organs accumulate predominantly ocotillol derivatives, which may account for 50% of the total ginsenoside content (Xia et al., 2022). Moreover, the quantitative ratio of saponins may differ among subspecies, resulting in differences in their pharmacological activity (Zhu et al., 2004; Van Le et al., 2015). The content and composition of ginsenosides including major ginsenosides Rb1, Rg1, and majonoside R2 also change with the plant’s age, reaching a maximum in 5-year-old plants, which makes this age optimal for harvesting (Vo et al., 2015; Vu-Huynh et al., 2020).

In the past decades, overexploitation of natural habitats, slow growth, and poor regeneration have led to a dramatic decline in the wild population of Vietnamese ginseng and put the species on the edge of extinction. In 2007, *P. vietnamensis* was listed in the Red Data Book of Vietnam (Vu et al., 2020). Systematic studies on the sexual and vegetative propagation of Vietnamese ginseng are being conducted to restore its population and expand plantation cultivation potential in Vietnam (Le et al., 2022). However, plantation cultivation of Vietnamese ginseng is associated with numerous difficulties due to the complex requirements of the species to the growth environment and a period of 5–7 years to achieve high commercial quality (Trong et al., 2017). There are also massive gaps in the mass production of *P. vietnamensis* using biotechnological approaches such as tissue, organ, and cell culture, particularly in regard to the regulated production of bioactive secondary metabolites, due to very few studies conducted on the topic so far. In particular, there is no consistency in the methodology used for induction and selection of the highly productive cell lines, and little is known about the main factors influencing ginsenoside accumulation in the tissue culture of this species.

For adventitious roots cultured in flasks, maximum growth and biosynthesis of majonoside R2, Rb1, and Rg1 were achieved on a modified MS medium with indole-3-butyric acid (IBA) and 6-benzylaminopurine (BA); ginsenoside accumulation could be increased further by elicitation (Nguyen et al., 2019). The same authors demonstrated that adventitious roots of *P. vietnamensis* could be successfully cultured in a 20-L barbotage bioreactor (Nguyen et al., 2019). Ha et al. (2016) used Schenk and Hildebrandt (SH) medium to develop several hairy root lines of *P. vietnamensis* with a specific growth rate reaching 0.049 day^−1^ and a doubling time of 14 days. The total fresh weight (FW) of cultured roots increased 19-fold after 90 days, significantly exceeding the productivity of field-cultured plants (60-g gain in 5 years). The total ginsenoside content in hairy roots was 70% of that in rhizomes of the 6-year-old plant (Ha et al., 2016). Later, the authors reported cultivating hairy roots in a 20-L bioreactor (Ha et al., 2019).

In plant cell cultivation, active growth of *P. vietnamensis* callus cultures was observed on SH medium with 2,4-dichlorophenoxyacetic acid (2,4-D) and thidiazuron under a 10-h photoperiod provided by fluorescent white light (Duong et al., 2011). In subsequent studies, increased callus growth intensity was demonstrated using LED lamps of different spectra (Duong et al., 2015). Sobolkova et al. used modified Gamborg (B5) and Murashige and Skoog (MS) media with different combinations of 2,4-D, 1-naphthaleneacetic acid (NAA), BA, and kinetin in darkness for callus induction followed by a selection of callus lines with high proliferation potential (Sobolkova et al., 2018).

Several studies have focused on optimizing *P. vietnamensis* cell suspension growth in flasks and bioreactors. Nguyen et al. demonstrated that increasing the concentration of sucrose (up to 50 g L^−1^) and macronutrients KNO_3_, MgSO_4_, and CaCl_2_ positively affected both the growth of the cell suspension culture in flasks and accumulation of ginsenosides (Nguyen et al., 2007, Nguyen et al., 2008). Successful cultivation of Vietnamese ginseng suspension cell culture in a 2-L bioreactor was achieved using MS medium with the synthetic auxin 3-benzo[*b*]-selenienyl acetic acid (BSAA) under constant illumination of 15 μmol/(m^2^·s) (Jacques et al., 2007). Another study reported good growth of the suspension cell culture in flasks filled with 1/2MS medium with 50 g L^−1^ sucrose and growth regulators 2,4-D, NAA, indole-3-acetic acid (IAA), and kinetin under 16-h light/8-h darkness photoperiod (Le et al., 2016). Yeast extract was effective as an elicitor (Trong et al., 2017). Although all authors achieved stable growth and ginsenoside production in cell cultures, their results are difficult to generalize due to very different combinations of medium formulations, growth regulators, light/dark conditions, and elicitation, and the main factors (or their combination) favoring cell culture productivity remain obscure.

Induction and selection of the superior suspension cell lines with intensive growth, broad ginsenoside spectrum, and a small-aggregated structure are the key steps toward advanced cell-based biotechnology. However, for many medicinal species including *P. vietnamensis*, this remains a challenging task. In the present study, we developed six lines of *P. vietnamensis* suspension cell culture and evaluated their growth, cytological, and ginsenoside profiles to select the most promising variants for further studies, particularly for bioreactor cultivation.

## 2 Materials and methods

### 2.1 Cell culture origin and cultivation conditions

**Figure 1:**
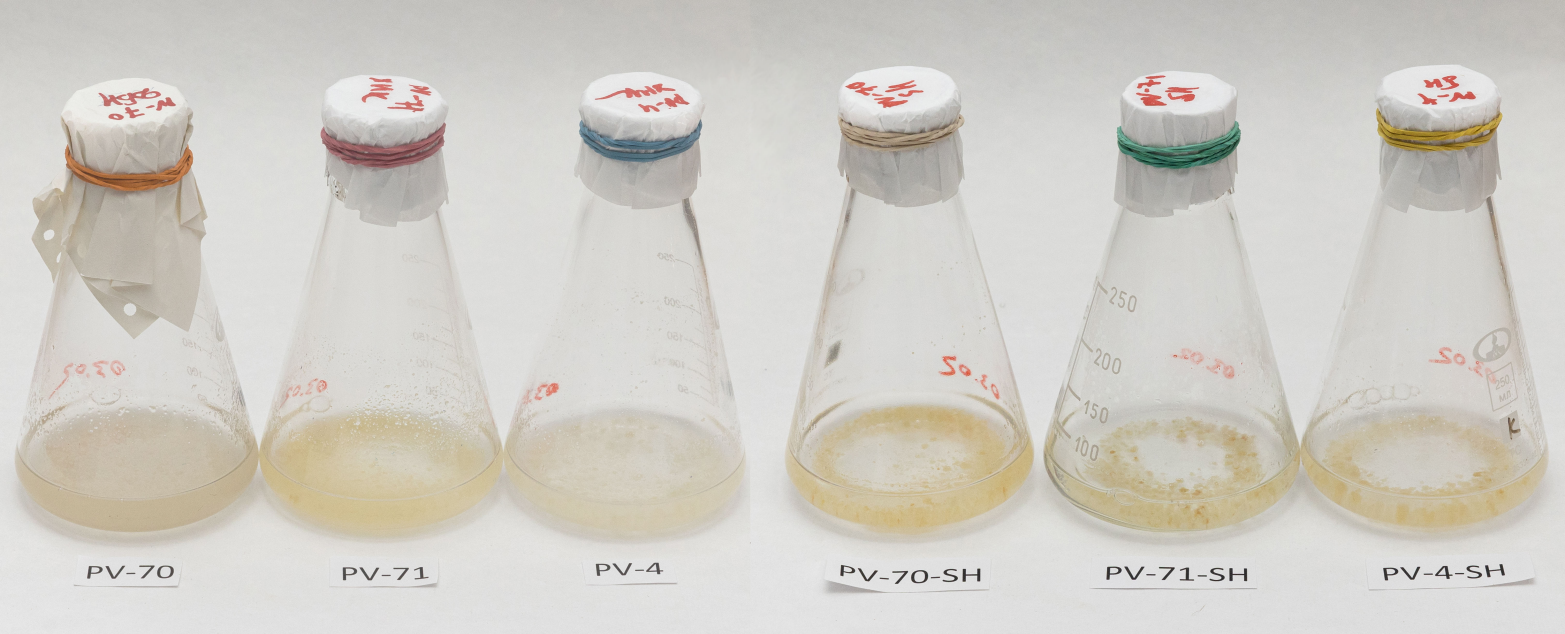
Six lines of the suspension cell culture of *Panax vietnamensis* were used in the study 3 years after the initiation. Cultures were photographed at the beginning of the exponential growth phase.

Callus cultures of *P. vietnamensis* Ha et Grushv. were developed at the K.A. Timiryazev Institute of Plant Physiology of the Russian Academy of Sciences (Moscow, Russia) from rhizome sections of a single plant identified and provided by the Central Botanical Garden NAS of Belarus as described earlier (Sobolkova et al., 2018). The multi-step process of medium optimization for callus induction and proliferation employed a total of 17 medium formulations differing in mineral salt base (MS, SH, or Gamborg), carbohydrate course (sucrose or glucose), vitamins, and combinations of growth regulators (2,4-D, NAA, BA, and kinetin) (**Supplementary Figure S1**). Nine callus lines originating from three different explants, PV-4, PV-70, and PV-71, were selected based on growth intensity and cultured in liquid media to form cell suspensions (**Supplementary Figure S1**). After 5 months, six cultures turned to stable and intensively growing suspension cell lines (**Figure 1**). The composition of media used for cell suspension cultivation is given in **Table 1**. The same numerical codes indicate cell lines developed from the same explants, i.e., cell lines PV-4 and PV-4-SH originate from one explant; PV-70 and PV-70-SH originate from another explant of the same rhizome; PV-71 and PV-71-SH originate from the third explant of the same rhizome.

**Table 1:** Medium composition for suspension cell cultures of *Panax vietnamensis* analyzed in this study.

Medium components			Suspension cell lines*		
			PV-70	PV-71 PV-4	PV-70-SH PV-71-SH PV-4-SH
			**Culture media**		
Mineral base			В5	В5	SH
Vitamins	Thiamine (В1)		1.0 mg L^–1^	1.0 mg L^–1^	5.0 mg L^–1^
	Pyridoxine (В6)		1.0 mg L^–1^	1.0 mg L^–1^	0.5 mg L^–1^
	Calcium pantothenate		5.0 mg L^–1^	5.0 mg L^–1^	−
	Nicotinic acid		−	−	5.0 mg L^–1^
Growth regulators	Auxins	2,4-D	1.0 mg L^–1^	0.5 mg L^–1^	1.0 mg L^–1^
		NAA	1.0 mg L^–1^	0.1 mg L^–1^	−
	Cytokinins	Kinetin	−	0.05 mg L^–1^	0.1 mg L^–1^
		BA	0.05 mg L^–1^	−	−
Sucrose			30 g L^–1^	30 g L^–1^	30 g L^–1^

B5, mineral salt base according to Gamborg et al. (1968) modified with Murashige and Skoog microelements (Murashige and Skoog, 1962); SH, macro- and microelements according to (Schenk and Hildebrandt 1972); 2,4-D, 2,4-dichlorophenoxyacetic acid; NAA, 1-naphthaleneacetic acid; BA, 6-benzylaminopurine; –, compound not used.

*Cell lines with the same numerical code originate from the same explant.

The selected six suspension cell lines were maintained in their respective media for 3 years before being analyzed in this study. Cultivation was performed in 250-mL Erlenmeyer flasks filled with 40 mL of liquid medium on a stationary orbital shaker at 90–100 rpm, 26°C–27°C, and 70%–75% relative air humidity in darkness. The inoculum density was 0.5 or 1.0 gDW L^−1^. Subcultures were performed every 25–30 days. In the experiments on the growth curve record, the culture period was extended to 40–45 days to capture the stationary and degradation phases.

### 2.2 Assessment of growth and physiological characteristics of the suspension cell cultures

To assess the growth and physiological state of the cell cultures, fresh and dry weights (DW) of cell biomass, level of cell aggregation, and cell viability were recorded every 2–3 days during the cultivation cycle (Titova et al., 2021). For each time point, three flasks (n = 3) were collected and analyzed.

To estimate the fresh weight, 10–15 mL aliquots of cell suspension were pipetted on paper filters, and the culture medium was removed under a vacuum; cell biomass was washed three times with distilled water under a vacuum and weighed. To estimate dry weight, cell biomass was dried to a constant weight at 40°C. The biomass samples for chemical analysis (see below) were collected and dried following the same procedure.

Cell viability was determined by staining with 0.1% Phenosafranin (3,7-diamino-5-phenylphenazinium chloride) (modified from Dixon and Gonzales, 1994) and expressed as the percentage of cell aggregates composed of colorless (living) cells out of total number of aggregates. For each experimental condition, at least 250 cell aggregates were examined in each of the three replicates.

Aggregation was defined as the ratio of aggregates comprising a certain number of cells. To increase the contrast, cell suspension samples were stained with 0.1% Phenosafranin, and the number of aggregates with different cell numbers was counted under a light microscope.

Photographs of cell aggregates on day 14 of the growth cycle (exponential growth phase) were taken using an Axio Imager Z2 microscope (Zeiss, Jena, Germany) with an Axio-Cam MR digital camera.

Growth parameters were calculated according to Nosov (2012) and Titova et al. (2021).

Specific growth rate at the exponential growth phase:

$µ={µ}_{\text{i}\_\text{max}}=\frac{\text{l}\text{n}\frac{{\text{X}}_{\text{i}}}{{\text{X}}_{\text{i}-1}}}{{\text{t}}_{\text{i}}-{\text{t}}_{\text{i}-1}}$ , [day^−1^].

Dry biomass doubling time:

$\text{τ}=\frac{\text{ln}2}{\text{μ}}$ , [day].

Productivity on dry biomass:

$\text{P}={\text{P}}_{\text{i}\_\text{max}}=\frac{{\text{X}}_{\text{i}}-{\text{X}}_{\text{i}-1\;}}{{\text{t}}_{\text{i}}-{\text{t}}_{\text{i}-1}}$ , [g (L·day)^−1^].

Economic coefficient:


$$\text{Y}=\frac{{\text{X}}_{\text{max}}-{\text{X}}_{0\;}}{{\text{S}}_{0}},$$

where X_i_ and X_i−1_ are dry cell biomass concentrations (g L^−1^) at time points t_i_ and t_i−1_, respectively; X_0_ and X_max_ are, respectively, initial and maximum dry cell biomass concentrations (g L^−1^); S_0_ is the initial sucrose concentration in the medium (g L^−1^).

### 2.3 Sample preparation for UHPLC–Orbitrap–MS/MS and UPLC–TOF–MS

#### 2.3.1 Chemicals and reagents

For sample preparation, high-performance liquid chromatography (HPLC)-grade methanol (Panreac, Barcelona, Castellar del Vallès, Spain) and ultrasonic bath (Saphir, Moscow, Russia) were used. The mobile phase was prepared using HPLC–UV-grade acetonitrile (Panreac, Barcelona, Castellar del Vallès, Spain), MS-grade formic acid (Fluka, Seattle, DA, USA), and water, which was prepared by Milli-Q deionization system (Millipore, Burlington, MA, USA). Standards of ginsenosides Rb2, Rb3, Rg3, Rc, Rb1, Rd, Re, Rf, Rh1, R1, R0 and pseudoginsenoside F11 with purity >98% were purchased from PhytoLab (Vestenbergsgreuth, Germany).

#### 2.3.2 Sample preparation

To analyze secondary metabolites, the cell biomass of six *P. vietnamensis* lines was dried at 40°C. Each sample in three biological replicates was used for analysis. Powdered material (10 or 40 mg) was placed in a micro-centrifuge tube and extracted by 2 mL of 70% (v/v) aqueous methanol in a UZV-12 ultrasonic bath (Sapfir, Saint Petersburg, Russia) at 35 kHz for 30 min at room temperature followed by centrifugation in a centrifuge PE-6926 EKROS (Ecochim, Moscow, Russia) at 15,000 *g* for 15 min. The extraction process was repeated three times. The resulting alcohol extracts of each sample were combined and evaporated under vacuum in an evaporating flask. Then, 900 µL of a 5% (v/v) aqueous acetic acid (Chimmed, Moscow, Russia) and 300 µL of 70% (v/v) aqueous methanol solution were sequentially added to the dry extract, ultrasonicated, transferred to micro-centrifuge tubes, and centrifuged at 15,000 *g* for 15 min. The supernatant was then transferred to micro-centrifuge tubes and evaporated in a Centrifuge Concentrator (Eppendorf, Hamburg, Germany). Then, each dry extract was dissolved in 1 mL of a 5% (v/v) aqueous acetic acid solution, and the mixture was passed through a Supelclean ENVI-18 solid-phase extraction (SPE) cartridge (Supelco, Bellefonte, PA, USA). The cartridge was washed with 5 mL of 5% (v/v) acetic acid solution; analytes were eluted with 300 µL of 96% ethanol three times, and the purified extracts were evaporated under vacuum in a Centrifuge Concentrator (Eppendorf, Germany). The resulting dry extracts were stored at −18°C until they were used for chemical analysis. Before the analysis using UPLC-TOF-MS, dry extracts were dissolved in 500 µL of 70% (v/v) aqueous methanol with 0.1% formic acid added solution using ultrasound.

For ultra-high-performance liquid chromatography (UHPLC)–Orbitrap–tandem mass spectrometry (MS/MS), ginsenoside identification was performed using the combined sample out of all six lines in three biological replicates. For this, 50 µL of each SPE purified extract obtained as described above was transferred to a 2-mL centrifuge tube and evaporated under vacuum in a Centrifuge Concentrator (Eppendorf, Germany). Then, the combined dry extract was dissolved in 100 µL of 80% (v/v) aqueous methanol with 0.1% formic acid added solution.

### 2.4 Ginsenoside identification using UHPLC–Orbitrap–MS/MS

#### 2.4.1 Data acquisition

Orbitrap Exploris 120 mass spectrometer combined with Vanquish UHPLC system (Thermo Fisher, Waltham, MA, USA) was used for sample profiling and fragmentation spectra acquisition. Chromatographic separation was performed on reversed-phase column Acclaim C18 2.2 µm (2.1 × 150 mm, Dionex, Sunnyvale, CA, USA) in gradient elution mode with 0.1% (v/v) solution of formic acid in water (solvent A) and a 0.1% (v/v) solution of formic acid in acetonitrile (solvent B) as a mobile phase at 0.4 mL min^–1^. Gradient parameters were as follows (B, % by volume): 0–20 min, 20%; 20–25 min, 20%–37%; 25–26 min, 37%–46%; 26–30 min, 46%–95%. The injection volume was 20 µL, the column oven temperature was 40°C, and the autosampler temperature was 4°C. Heated electrospray ionization (ESI) source parameters were as follows: negative ionization; the ion transfer voltage was −2,500 V, the sheath/auxiliary/sweep gas was 50/10/1 arb, the ion transfer temperature was 325°C, and the vaporizer temperature was 350°C. Scan parameters were as follows: the resolution was 30,000 (for both MS^1^ and MS^2^ spectra), the scan range was 200–2,000 *m/z*, the RF lens was 70%, the number of microscans was 3, and the data type was centroid. MS^2^ spectra were acquired in data-dependent acquisition (DDA) mode with a target “*m/z* - retention time” list containing major signals found during profiling data analysis. Higher-energy collisional dissociation (HCD) fragmentation parameters were as follows: isolation window was 1 *m/z* with isolation offset turned off; normalized collision energy was 30%, 45%, 60%, and 70%; normalized automatic gain control (AGC) target was 200% with a maximum injection time parameter set to “auto”.

#### 2.4.2 Data processing

Mass spectra analysis was performed using FreeStyle (1.8 SP2, Thermo Fisher, Waltham, MA, USA) with built-in tools for elemental composition annotation. Identification of terpene glycosides was performed by comparing their fragmentation patterns with those of commercial standard mixtures.

### 2.5 Quantitative analysis of ginsenosides using UPLC–TOF–MS

#### 2.5.1 Data acquisition

Ultra-performance liquid chromatography coupled with electrospray ionization mass spectrometry using a Waters Acquity UPLC chromatograph (Waters, Milford, MA, USA) equipped with a XEVO QTOF hybrid quadrupole time-of-flight mass spectrometer (Waters, USA) was used for sample profiling. The analysis was performed in negative ion mode in the *m/z* range of 550–1,500. The injection volume was 5 µL. The ionization source parameters were as follows: ionization source temperature was 150°C, desolvation temperature was 600°C, capillary voltage was 3 kV, sample injection cone voltage was 40 V, and nitrogen (desolvation gas) flow rate was 1,000 L h^–1^. Chromatographic separation conditions were as follows: Acquity UPLC BEH C18 (50 × 2.1 mm, 1.7 μm, Waters, USA) was used with column temperature 40°C, and mobile phase flow rate was 0.4 mL min^–1^. A 0.1% (v/v) solution of formic acid in water (solvent A) and a 0.1% (v/v) solution of formic acid in acetonitrile (solvent B) were used as the mobile phase. Chromatographic separation was performed using a gradient elution mode. The composition of the mobile phase changed according to the following program (B, % by volume): 0–2 min, 20%; 2–20 min, 20%; 20–20.10 min, 20%; 20.10–25.00 min, 20%–37%; 25.00–26.00 min, 37%–46%; and 26.00–26.10 min, 46%–95%.

#### 2.5.2 Data processing

The ginsenoside peak area was integrated into MassLynx software (Waters, USA). The mass chromatogram window was 50 ppm. Integral peak area quantification was performed for malonyl ginsenoside Rb1, malonyl ginsenoside Rb1 isomer, malonyl ginsenoside Rb2/Rb3, malonyl ginsenoside Rb2/Rb3 isomer, ginsenoside R0, ginsenoside R0 isomer, and chikusetsusaponin IV/isomer (1, 2, 3, and 4 + 5) as [M–H]^–^ ions and for vinaginsenoside R1 and ginsenoside Rb1 as [M–H+FA] ^–^ ions; calculations were performed based on commercial standards of ginsenosides (Rb1, Rb2/Rb3, R0, and pseudoginsenoside F11) of the same chemical group.

### 2.6 Statistical analysis of the data

Statistical analysis of the results was performed using Excel (MS Office 2016) and GraphPad Prism 8.0.1 programs. Data are presented as the mean values and standard deviations recorded for the triplicates (three flasks or three fixed-size cell suspension samples) for each data point or variant. For viability measurement, suspension samples were taken from three flasks of the same variant, and at least 250 cell aggregates were examined in each sample. For aggregation measurements, two or three suspension cell samples were collected from each of the three flakes for the same cell line. Statistical significance of differences was estimated using ANOVA followed by Duncan’s Multiple Range test or Kruskal–Wallis one-way analysis of variance at p< 0.05.

## 3 Results

### 3.1 Suspension cell culture development and growth

Suspension cell cultures of *P. vietnamensis* Ha et Grushv. were induced from the three most intensively growing callus lines originating from different segments of a single rhizome as described earlier (Sobolkova et al., 2018) (see also **Materials and Methods**, **Supplementary Figure S1**). Callus lines were placed in liquid media to initiate cell suspensions (**Supplementary Figure S1**). From these variants, after 5 months of selection, six suspension cell lines, PV-4, PV-4-SH, PV-70, PV-70-SH, PV-71, and PV-71-SH, demonstrated stable intensive growth and were further maintained on their respective media for 3 years before being evaluated in this study (**Table 1**, **Figure 1**).

**Figure 2:**
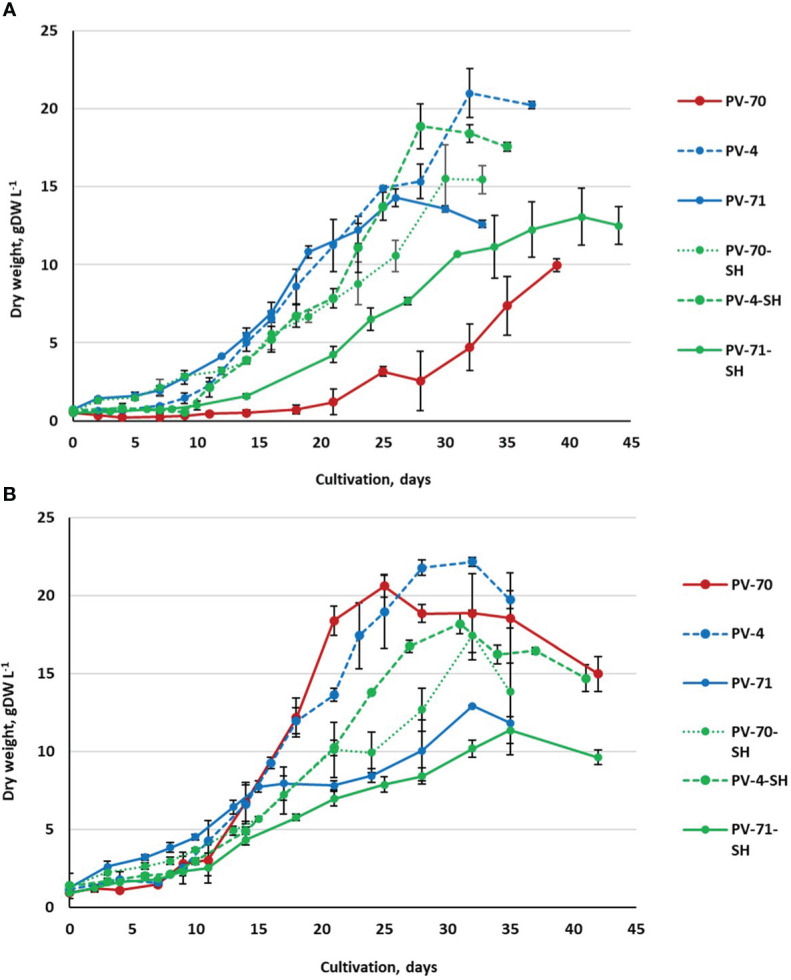
Growth curves (based on dry weight) of the suspension cell cultures of *Panax vietnamensis*, lines PV-70, PV-71, PV-4, PV-70-SH, PV-71-SH, and PV-4-SH, during cultivation in 250-mL flasks using two inoculum densities: **(A)** *X*_0_ = 0.5 gDW L^−1^ and **(B)** *X*_0_ = 1.0 gDW L^−1^. Growth curves of the same color correspond to cell lines cultured in media of the same composition. For every cell line, data are presented as mean values from three flasks (*n* = 3) and standard deviations for each data point.

In the first series of experiments, the growth dynamics of the six suspension cell lines were studied in the course of cultivation in 250-mL flasks at two inoculum sizes, *X*_0_ = 0.5 gDW L^–1^ and *X*_0_ = 1.0 gDW L^−1^. Growth curves of the cell lines based on DW are shown in **Figure 2**. More detailed growth curves, including the dynamics of the FW and cell viability, are presented in **Supplementary Figure S2**. Growth parameters calculated based on DW are given in **Table 2**.

**Table 2:** Growth parameters of six suspension cell lines of *Panax vietnamensis* during cultivation in 250-mL flasks with inoculum density (*X*_0_) of 0.5 or 1.0 gDW L^−1^.

Cell line	Auxin/cytokinin *	*μ*, day^−1^	*τ*, day	*Xmax*, gDW L^−1^	*Y*	*Pmax*, g (L·day)^−1^
*Inoculum density X_0 _= 0.5 gDW L^−1^*						
**PV-70**	D 1+N 1/B 0.05	0.13 ± 0.01	5.5 ± 0.1	9.96 ± 0.39	0.31 ± 0.01	0.24 ± 0.01
**PV-4**	D 0.5+N 0.1/K 0.05	0.21 ± 0.01	3.3 ± 0.1	21.01 ± 1.57	0.68 ± 0.05	0.64 ± 0.05
**PV-71**	D 0.5+N 0.1/K 0.05	0.14 ± 0.01	4.8 ± 0.1	14.29 ± 0.57	0.45 ± 0.02	0.53 ± 0.02
**PV-70-SH**	D 1/K 0.1	0.11 ± 0.01	6.6 ± 0.3	15.80 ± 0.50	0.51 ± 0.02	0.50 ± 0.07
**PV-4-SH**	D 1/K 0.1	0.23 ± 0.01	2.7 ± 0.2	18.88 ± 1.44	0.61 ± 0.05	0.65 ± 0.05
**PV-71-SH**	D 1/K 0.1	0.13 ± 0.01	5.2 ± 0.2	13.08 + 1.83	0.42 ± 0.06	0.33 ± 0.01
*Inoculum density X_0 _= 1.0 gDW L^−1^*						
**PV-70**	D 1+N 1/B 0.05	0.20 ± 0.03	3.4 ± 0.3	20.60 ± 0.73	0.66 ± 0.02	0.83 ± 0.04
**PV-4**	D 0.5+N 0.1/K 0.05	0.19 ± 0.01	3.7 ± 0.1	22.14 ± 0.29	0.70 ± 0.01	0.74 ± 0.02
**PV-71**	D 0.5+N 0.1/K 0.05	0.10 ± 0.01	6.9 ± 0.2	12.92 ± 0.06	0.39 ± 0.01	0.43 ± 0.02
**PV-70-SH**	D 1/K 0.1	0.09 ± 0.01	7.5 ± 0.3	17.45 ± 1.58	0.54 ± 0.05	0.50 ± 0.05
**PV-4-SH**	D 1/K 0.1	0.12 ± 0.01	5.9 ± 0.3	18.17 ± 0.64	0.56 ± 0.02	0.57 ± 0.01
**PV-71-SH**	D 1/K 0.1	0.11 ± 0.01	6.2 ± 0.1	11.36 ± 0.87	0.35 ± 0.03	0.30 ± 0.02

All parameters were calculated based on dry weight.

*Xmax*, maximum dry cell weight; *μ*, specific growth rate at exponential growth phase; *τ*, doubling time; *Y*, economic coefficient; *Pmax*, productivity based on dry weight.

*Numbers after abbreviations indicate concentrations of growth regulators, e.g., D 0.5 and D 1 correspond to 0.5 and 1.0 mg L^–1^ of 2,4-D, respectively; K 0.05, K 0.1, and K 1 correspond to 0.05, 0.1, and 1 mg L^–1^ of kinetin, respectively; N 0.1 and N 1 correspond to 0.1 and 1.0 mg L^–1^ of NAA, respectively.

All cell suspension lines showed satisfactory growth and productivity with 0.1–0.2 day^−1^ specific growth rate, economic coefficient of 0.31–0.70, and productivity on dry biomass of 0.30–0.83 gDW (L·day)^−1^. The highest values of economic coefficient, maximum biomass accumulation, and specific growth rate were recorded for lines PV-4, PV-70, and PV-4-SH (**Table 2**).

Most lines, except for PV-70, grew better at the inoculum of 0.5 gDW L^−1^. Only in PV-70 did a subculture with an inoculum of 1.0 gDW L^−1^ lead to an increase of 1.5–2.0 times in specific growth rate, dry biomass accumulation, and economic coefficient and an increase of 3.5 times in productivity (**Table 2**). The effect of the medium on growth parameters was inconsistent, without a clear trend.

The growth phases determined based on a growth curve plotted in semi-logarithmic coordinates (**Supplementary Figure S3**, **Supplementary Table S1**) also showed no clear pattern depending on the medium type or inoculum and were mainly line-specific. The lag phase and exponential growth phase varied among the lines as well as for the same line at different inoculums. The most extended lag phase was observed for line PV-70. Cell line PV-71-SH required the longest time (over 35 days) to reach maximum biomass accumulation (X_max_) (**Figure 2**). For other lines, the time to achieving *X*_max_ varied within 26–32 days at *X*_0_ = 0.5 gDW L^−1^ and within 25–32 days at *X*_0_ = 1.0 gDW L^−1^. PV-70 and PV-71-SH showed the lowest viability in the exponential growth phase, while other cell lines retained over 80% viability at the exponential growth phase (**Supplementary Figure S2**). All lines had a short stationary phase, if any, and the degradation phase started almost immediately after achieving a maximum dry weight. For some cell lines, the beginning of stationary and degradation phases varied between individual flasks, resulting in notable deviations in the ratio of fresh/dry weights and cell viability between replications. These growth variations were likely due to the heterogeneity of the cell populations.

### 3.2 Cytological characteristics of the suspension cell cultures

**Figure 3:**
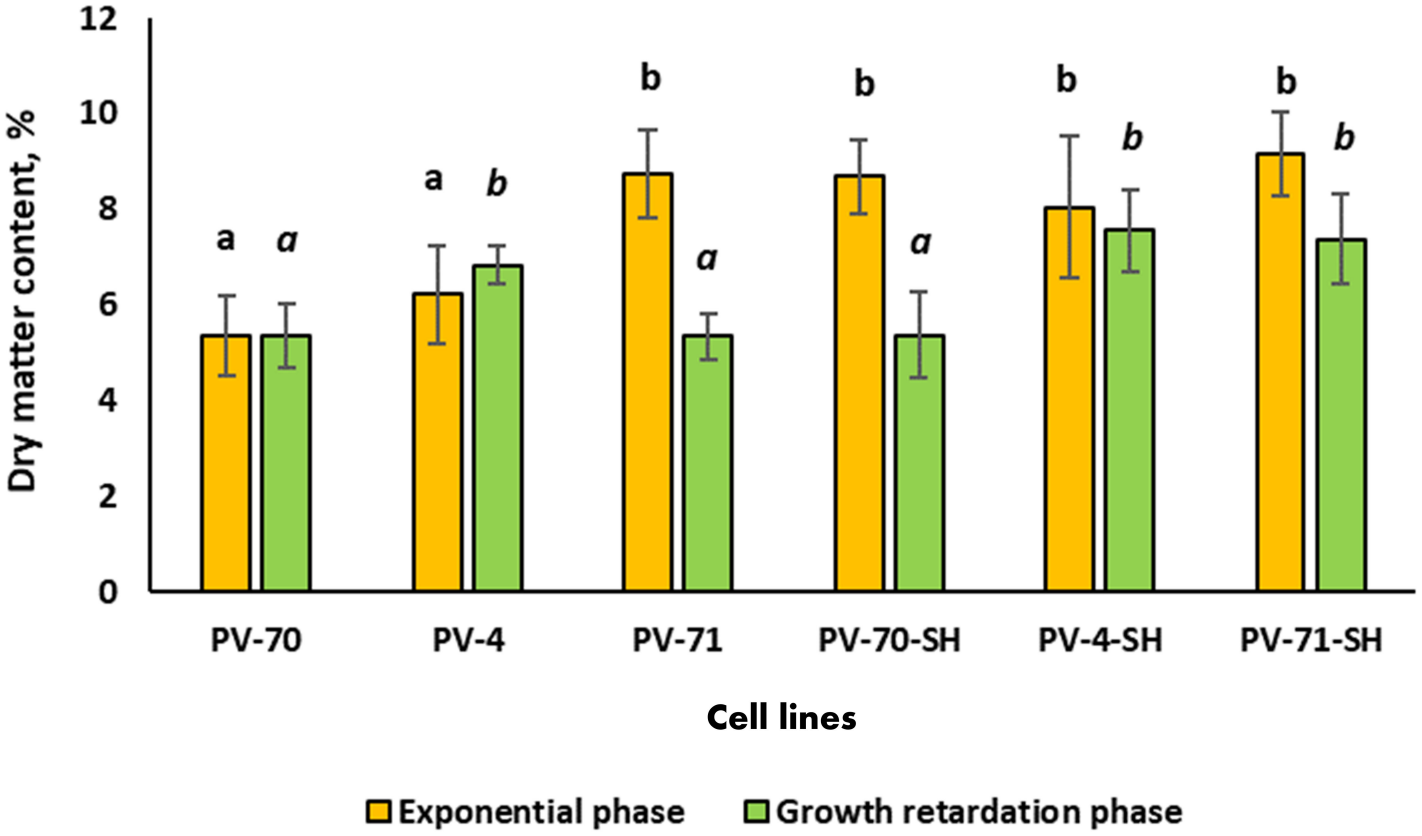
Dry matter content of the suspension cell culture of *Panax vietnamensis* in the middle of exponential phase and growth retardation phase. Values labeled by the same letter are not significantly different at *p*< 0.05 according to the Kruskal–Wallis test (n = 10–30 measurements).

The dry matter and cell water contents were determined in the middle of exponential and growth retardation phases for all lines subcultured at the optimum inoculum size found in the previous experiment (**Figure 3**). At the exponential growth phase, the maximum dry matter content (8% and higher) and the minimum water content were observed for lines PV-71, PV-70-SH, PV-71-SH, and PV-4-SH (**Figure 3**). At the growth retardation phase, the dry matter content decreased by 40% in the cell lines PV-71 and PV-70-SH and by 20% in the line PV-71-SH. For the other lines, the changes in cell water content during the cultivation cycle were insignificant. As for growth parameters, there was no apparent connection between medium composition and water content in the cells of different lines.

**Figure 4:**
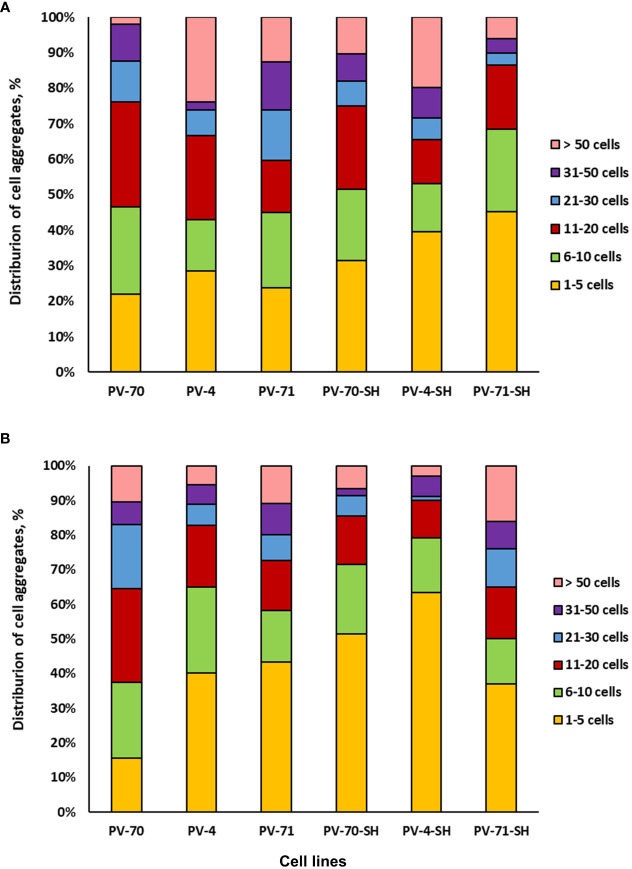
Aggregation of six suspension cell lines of *Panax vietnamensis*: **(A)** middle-exponential phase and **(B)** growth retardation phase (n = 6–9 measurements).

Cell aggregation level and cell size are important culture characteristics, as they may favor or hamper the cultivation of cell suspension in bioreactors (Gaid et al., 2017). Hence, in the present study, the six suspension cell lines of *P. vietnamensis* were analyzed for the level of cell aggregation at the exponential and growth retardation phases. As demonstrated in **Figure 4**, over 80% of aggregates in all lines were relatively small and composed of less than 50 cells. In lines PV-70-SH, PV-71-SH, and PV-4-SH, over 50% of aggregates consisted of 1–10 cells. Line PV-70 was the most aggregated, with over 50% of aggregates having 11–50 cells. In general, cell lines growing on SH medium with 1 mg L^–1^ of 2,4-D and 1 mg L^–1^ of kinetin had a more significant proportion of small-size aggregates (up to 10 cells), but this difference was not statistically sound.

Photographs of the six lines of *P. vietnamensis* at the exponential growth phase are given in **Supplementary Figure S4**. Lines PV-4, PV-70, and PV-4-SH had many aggregates in the form of cell chains. Line PV-70 was the most heterogeneous regarding the shape of cells and aggregates. Line PV-71 was the most homogeneous, with rounded aggregates predominating in the population. Lines PV-71, PV-71-SH, and PV-4-SH were primarily characterized by small cells below 50 µm in diameter.

### 3.3 Profiling of ginsenosides in the suspension cell cultures of *P. vietnamensis*

Identification of triterpene glycosides of the suspension cell cultures of *P. vietnamensis* was performed at the time point corresponding to maximum biomass accumulation using a combination of the reversed-phase UHPLC–Orbitrap–MS/MS and ultra-performance liquid chromatography–time of flight–mass spectrometry (UPLC–TOF–MS). These methods enable fast and accurate identification of the compounds of plant origin by analyzing exact *m/z* values of protonated and deprotonated molecular ions, adduct ions, and fragment ions with the implementation of isotopic distribution analysis (Sun et al., 2017).

Identification of individual components was performed by comparing the experimental mass spectra and retention information obtained in this study with *m/z* values and retention times of individual commercial standards and literature data (Li et al., 2010; Yang et al., 2012, Yang et al., 2013). The negative ion registration mode was selected, as this regime provides the highest ginsenoside signal intensity and a more informative fragmentation pattern (Fuzzati et al., 1999).

**Figure 5:**
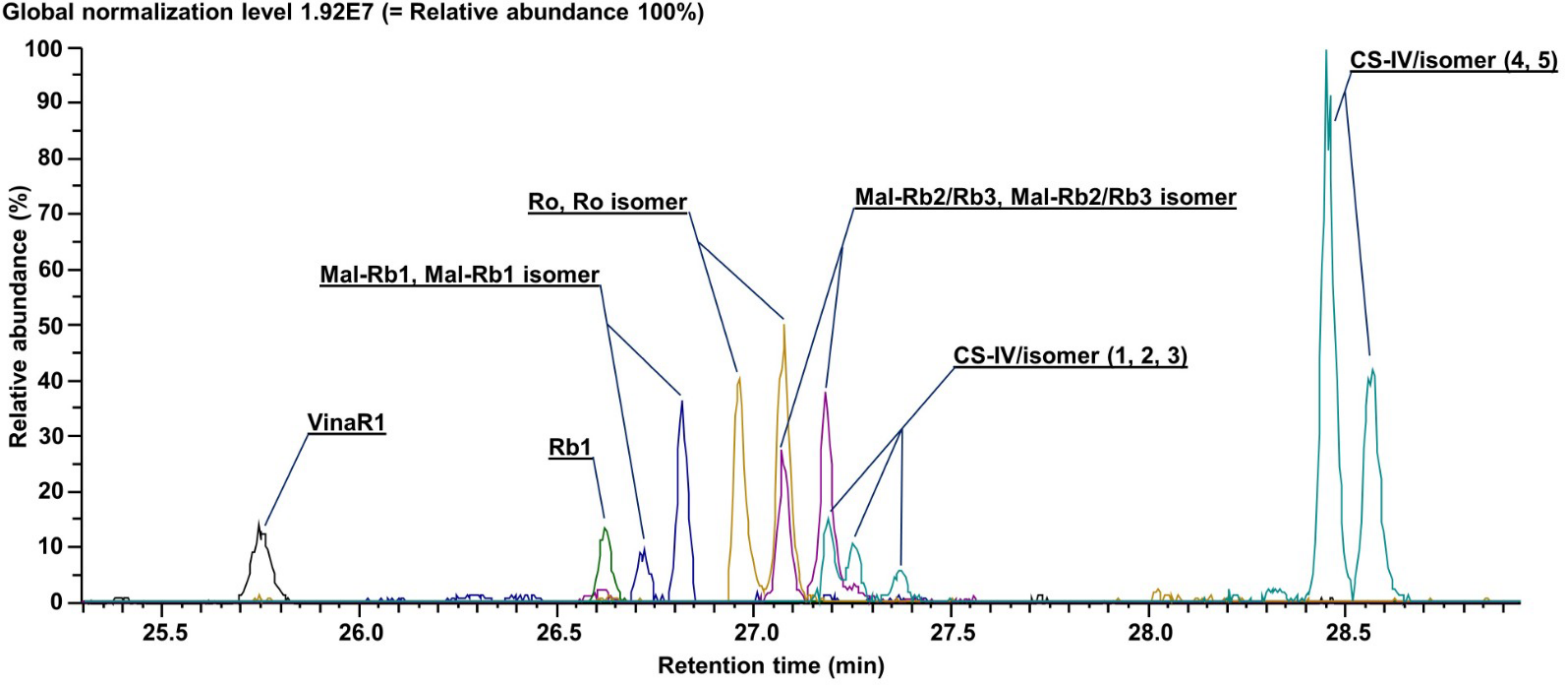
Extracted ion current chromatograms (total ion current) for annotated metabolites. Data were obtained using ultra-high-performance liquid chromatography–Orbitrap–tandem mass spectrometry (UHPLC–Orbitrap–MS/MS).

**Figure 5** shows extracted ion chromatograms (XIC) for the 13 major ginsenosides, their malonyl derivatives, and isomers in the combined sample of all six *P. vietnamensis* suspension cell culture lines. It should be noted that we were able to achieve the baseline separation for most of the analytes. By the analysis of fragments in tandem mass spectra (MS/MS spectra of individual ginsenosides are represented in **Supplementary Figures S6, S7**), accurately measured mass, and the retention time on the column (*t_r_*), we were able to identify all 13 compounds (**Supplementary Table S2**). Cell cultures contained ginsenosides of the protopanaxadiol group (Rb1, Rb2/Rb3, their malonyl derivatives, and isomers), ocotillol group (Vinaginsenoside R1), and oleanolic acid group (R0, R0 isomer, and isomers of chikusetsusaponin IV), while ginsenosides of protopanaxatriol group were not detected.

**Figure 6:**
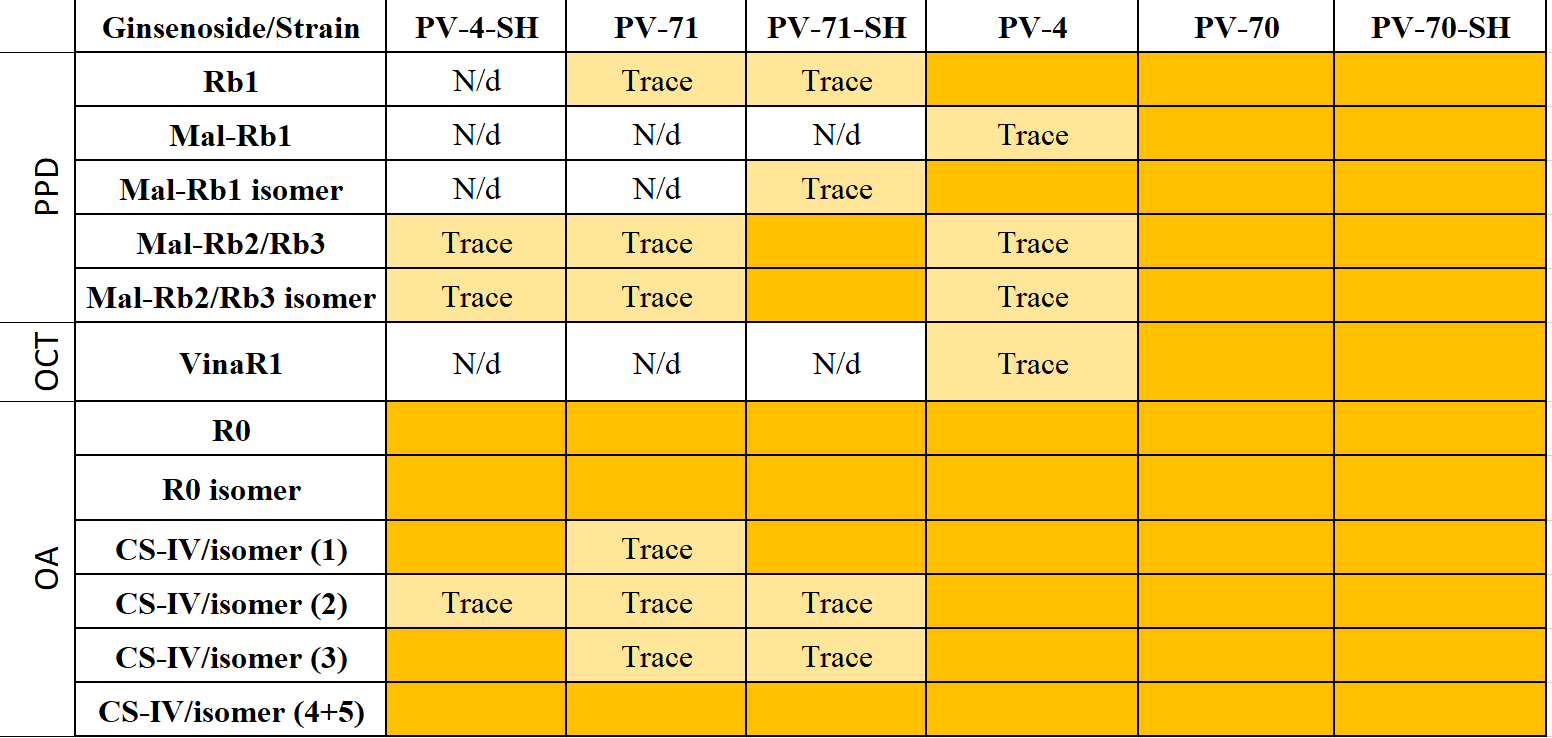
The presence of individual ginsenosides in the cell biomass of six suspension cell lines of *Panax vietnamensis*. Colored cells indicate ginsenosides detected in the sample. Trace, compounds found in trace amounts (peak area below 1,000 AU according to **Supplementary Table S3**); N/d, compound not found; PPD, protopanaxadiol group; OCT, ocotillol group; OA, oleanane group; VinaR1, vinaginsenoside R1; Mal, malonylated ginsenosides; CS-IV, chikusetsusaponin IV.

For semi-quantitative analysis of ginsenosides in individual *P. vietnamensis* cell lines, we analyzed cell extracts by UPLC–TOF–MS. Total ion current (TIC) chromatograms of the methanolic extracts of six *P. vietnamensis* cell culture lines are presented in **Supplementary Figure S5**, and the peak areas of individual compounds for each cell line are presented in **Supplementary Table S3**. The results of the qualitative analysis of ginsenosides in individual cell lines are presented in **Figure 6**.

It is clearly visible that the ginsenoside profiles are cell line-specific. At the same time, the culture medium can have a “personalized effect”—an influence on the production of some individual ginsenosides in separate lines (**Figure 6**). Cell suspension lines PV-70, PV-70-SH, and PV-4 contained all ginsenosides identified in this study including ginsenosides of the PPD, ocotillol, and oleanane groups. Vinaginsenoside R1 was present in the cell biomass extract of PV-70-SH, PV-70, and PV-4 but was not found in lines PV-71, PV-71-SH, and PV-4-SH. Ginsenoside Rb1 and its malonylated form and isomer were absent in PV-4-SH.

## 4 Discussion

### 4.1 Cell culture growth

In this study, suspension cell cultures of *P. vietnamensis* originating from different sections of a single rhizome and passed through a multi-step process of medium optimization were maintained for over 3 years on media with different mineral salt base and auxin/cytokinin ratios. These cell lines were assessed for growth and cytological characteristics as well as for ginsenoside composition to select the lines with higher potential for bioreactor cultivation.

Our results demonstrated that suspension cell cultures of *P. vietnamensis* maintained for over 3 years in 250-mL flasks retained good growth, indicated by a relatively high specific growth rate, maximum biomass accumulation, productivity on dry weight, and economic coefficient. This confirmed that the lines were well adapted to cultivation in their respective media. Most cell lines, except for PV-70, grew better at the lower inoculum of 0.5 gDW L^−1^, evidencing their high proliferation abilities. The effect of the medium including mineral salt base and hormone balance (auxin/cytokinin ratio, cytokinin type, elevated concentration of 2,4-D, and exclusion of NAA from the medium) was insignificant. The cell line provenance was, in general, more influential, resulting in similar growth parameters between cell lines originating from the same explant/primarily callus within the same inoculum density group, although this influence was not straightforward either. Overall, the recorded values of the growth parameters were higher than those reported for *P. vietnamensis* suspension cell cultures in other studies. For example, the highest DW accumulation during cultivation in flasks in modified MS medium was 12–13 gDW L^−1^ (Jacques et al., 2007), 10.3 gDW L^−1^ (Nguyen et al., 2007), and 3.2 gDW L^−1^ (Trong et al., 2017) compared to 10–22 gDW L^−1^ achieved in the present study.

The productivity of the suspension cell cultures on DW achieved in our study was higher compared to that of hairy and adventitious root cultures in flasks and bioreactors (Ha et al., 2016; Nguyen et al., 2019), plantation-grown plants, and plants collected from the wild (Vu-Huynh et al., 2020). In addition, for cells cultured in flasks, the maximum biomass accumulation could be reached in a relatively short time of 25–35 days. In contrast, plant rhizomes collected *ex vitro* should be at least 4–5 years old to be qualified as a commercial product; during this period, rhizome weight increased from 0.19 g to 10.29 g per plant (Vu-Huynh et al., 2020). For the adventitious root culture of *P. vietnamensis* in the modified MS medium, biomass production of approximately 15.6 gDW L^−1^ was recorded after 56 days (Nguyen et al., 2019).

### 4.2 Ginsenoside profiling in cell culture

An essential characteristic of the cell culture is the ability to retain the synthesis of secondary metabolites specific to the donor plant. A variety of analytical methods are currently available for analyzing metabolic profiles in ginseng and its commercial products: high-performance thin-layer chromatography (HPTLC) (Dallenbach-Toelke et al., 1987; Vanhaelen-Fastré and Faes, 2000), capillary electrophoresis (Tian et al., 2009), gas chromatography and gas chromatography–mass spectrometry (GC/GC-MS) (Cui, 1995; Park et al., 2018), nuclear magnetic resonance (NMR) spectroscopy (Yang et al., 2014), and HPLC/UPLC (Fuzzati, 2004; Park et al., 2013; Rodin et al., 2013). For routine analysis of ginseng products, HPLC/UHPLC methods, particularly with mass detection, are the most widely used due to their higher sensitivity and resolution and comprehensive profiling of ginsenosides (Rodin et al., 2013; Hsu et al., 2022). High-resolution mass spectrometers such as quadrupole time of flight (QTOF) and Orbitrap enable fast identification of ginsenosides with high mass accuracy even without commercial standards (Rodin et al., 2013; Sun et al., 2017).

In this study, ginsenosides in the suspension cell culture of *P. vietnamensis* were identified using UPLC–Orbitrap–high-resolution mass spectrometry and then quantified using UPLC–TOF–MS. A combination of the two methods based on mass detection is preferable for detecting ocotillol-type saponins because they lack chromophores in their structures, which makes the use of ultraviolet detection less effective (Nguyen et al., 2021). It is worth noting that ocotillol-type saponins having a tetrahydrofuran ring and a dammarane skeleton are rarely found in plants and may serve as specific markers for *P. vietnamensis* products (Le et al., 2018).

The composition of ginsenosides in cells was evaluated at the time point of achieving maximum biomass concentration for each line, based on previous observations that in the cell cultures of *Panax* spp., maximum accumulation of ginsenosides usually coincides with maximum biomass gain (Kochan and Chmiel, 2011; Le et al., 2019). For example, in *Panax japonicus* cell culture, the total content of ginsenosides peaked at late-exponential and stationary growth phases, corresponding to the lowest cell proliferative activity, cell elongation, and nuclear DNA reduplication (Smolenskaya et al., 2007a; Glagoleva et al., 2022).

The biochemical analysis resulted in the identification of ginsenosides of the PPD (Rb1, Rb2/Rb3, malonyl-Rb1, and malonyl-Rb2/Rb3), OA (R0 and chikusetsusaponin IV), and OCT (vinaginsenoside R1) groups and their isomers in cell biomass of *P. vietnamensis* suspension cultures (**Supplementary Table S2**). These results correspond well with published data on the ginsenoside composition in plants and *in vitro* cultures of *P. vietnamensis*. For example, accumulation of ginsenoside Rb1 was reported in plant rhizomes of Vietnamese ginseng as well as in hairy roots, adventitious roots, callus, and plantlets *in vitro* (Duong et al., 2015; Ha et al., 2016; Nguyen et al., 2019). Chikusetsusaponin V (R0) and Rb2/Rb3 were identified in plant rhizomes and roots (Kazuo, 2000). Vinaginsenoside R1 was detected in the underground plant parts (Nguyen et al., 1993; Le et al., 2018). Interestingly, in our study, isomers of chikusetsusaponin IV (OA-type) were identified in all cell lines. This ginsenoside is found predominantly in *P. japonicus* (Han et al., 2005; Wang et al., 2021), whereas its presence in *P. vietnamensis* has not been previously reported. It is also interesting that none of the cell cultures contained ginsenosides of the protopanaxatriol group that are often found in plants and plant cell cultures of various ginseng species (Nguyen et al., 1993, Nguyen et al., 2007; Le et al., 2018; Glagoleva et al., 2022).

The highest structural diversity of triterpene glycosides was observed for lines PV-70, PV-70-SH, and PV-4, where all identified ginsenosides were represented (**Figure 6**). OA-type ginsenosides were present in the biomass of all lines examined, whereas vinaginsenoside R1 (ocotillol group) was found only in lines PV-70, PV-70-SH, and PV-4.

Ginsenoside profiles of cell lines could be linked with their cytological features. The most limited spectrum of ginsenosides was observed in lines PV-71, PV-71-SH, and PV-4-SH. These cell lines were predominantly composed of tiny meristematic cells below 50 µm in diameter (**Supplementary Figure S4**). Earlier studies demonstrated that the accumulation of ginsenosides in ginseng cell cultures was closely associated with large parenchyma-like cells (Smolenskaya et al., 2007b) that in our study were predominant in PV-70 and PV-70-SH populations where all 13 compounds were found.

The balance of plant growth regulators in the medium may be another factor influencing ginsenoside production; however, literature data on the effect of hormonal composition on the cell growth and accumulation of ginsenosides in the *in vitro* cultures of *Panax* spp. are somewhat contradictory. The duration of experiments is usually limited to one or two subcultivations, and it is not always clear if the reported observations persist over time. In addition, most studies are performed on relatively “young” and, hence, unstable cell cultures less than one year after induction. Ginsenoside accumulation in cell cultures could be stimulated or suppressed in response to the same growth regulators depending on culture conditions. For example, Trong et al. (2017) investigated the effect of various concentrations of kinetin, BA, and NAA on young suspension cultures of *P. vietnamensis*, just initiated from the callus. Cultures were exposed to different growth regulators for 1 month. All growth regulators tested caused an increase in the accumulation of cell biomass, but the impact of each varied depending on the hormone type, concentration, and incubation period. Similarly, in young suspension cell cultures of *Panax ginseng*, maximum biomass accumulation was recorded in the medium with 2,4-D, while ginsenoside yield was higher in the medium containing either NAA or IBA (Lian et al., 2002). Ginsenoside biosynthesis could be further enhanced by adding cytokinin BA with no change in cell growth (Lian et al., 2002). The opposite effect of NAA and kinetin on the accumulation of ginsenosides was observed for the suspension culture of *P. ginseng* cells (Furuya et al., 1983). Other authors reported the stimulative effect of 2,4-D and NAA on ginsenoside synthesis in *Panax notoginseng* cell cultures (Zhong, 1998). However, in cell suspension cultures of *Panax quinquefolium*, the content of ginsenosides was remarkably decreased in the presence of 2,4-D (Zhong et al., 1996). Our data demonstrated that cell suspensions of *P. vietnamensis* had independent individual responses to the different hormonal compositions of the medium. Moreover, after 3 years of cultivation, the ginsenoside profile was mainly affected by the line origin rather than the culture medium. As a result, the effect of replacing growth regulators or changing the ratio of auxins and cytokinins may have different effects (up to the opposite) in different cell lines. Based on literature data, we may assume that the limited ginsenoside spectrum in PV-4-SH compared to its “pair” line PV-4 derived from the same callus may be associated with higher 2,4-D content in a nutrient medium, but these assumptions require further verification.

DNA methylation, which may be accumulated over time in cell culture due to multiple subcultures and constant proliferation, is also believed to be one of the factors responsible for alterations in the biosynthesis of secondary metabolites (Dubrovina and Kiselev, 2016; Sanchez-Muñoz et al., 2019). Such effects have been demonstrated for cell cultures of *P. ginseng* and *Taxus media* (Kiselev et al., 2013; Escrich et al., 2022). Some authors stressed that auxins in the nutrient medium might increase DNA methylation frequency in plant cells (Gao et al., 2014; Grzybkowska et al., 2020). When carrot embryogenic cell culture was transferred from 0.5 to 5 mg/L of 2,4-D in a culture medium, the level of 5-methyl-cytosine in the cells rose from 16% to 70% of total cytosine (Lo Schiavo et al., 1989). The level of DNA methylation during callus induction of *Malus xiaojinensis* was elevated in response to high 2,4-D concentration in the medium but reduced significantly with increasing BA concentration (Huang et al., 2012). Other authors stressed that there was no direct correlation between the content of 2,4-D in the culture induction medium and the methylation level (Gao et al., 2014). Yamamuro et al. (2016) highlighted that auxins in plant cells may act at different levels, inducing the interconnected processes of histone modification, DNA methylation, and microRNA modifications.

In conclusion, the effect of medium and growth regulators on growth and ginsenoside profiles observed in our study for *P. vietnamensis* cell suspensions were line-specific and inconsistent among cell lines. Such an effect may be attributed to individual characteristics of the lines acquired at the earlier stages of culture initiation, which requires further investigation.

## 5 Conclusions

Six lines of *P. vietnamensis* suspension cell cultures originating from the segments of a single rhizome and maintained for 3 years in different media demonstrated good growth during cultivation on flasks with a specific growth rate of 0.1–0.2 day^−1^, economic coefficient of 0.31–0.70, productivity on DW of 0.30–0.83 gDW (L·day)^−1^, and maximum biomass accumulation varying from 10 to 22 gDW L^−1^. Five out of six lines grew better when subcultured at the inoculum density of 0.5 gDW L^−1^ compared to 1.0 gDW L^−1^. Thirteen ginsenosides, their derivatives, and isomers were identified in cell biomass extracts using UPLC–Orbitrap–MS/MS including Rb1, Rb2/Rb3, malonyl-Rb1, and malonyl-Rb2/Rb3 (protopanaxadiol group), R0 and chikusetsusaponin IV (oleanane group), and vinaginsenoside R1 (ocotillol group). To the best of our knowledge, isomers of chikusetsusaponin IV were found and identified in cell cultures of *P. vietnamensis* for the first time. It is proposed that the major differences in biosynthetic profiles between the cell lines may be attributed to differences acquired at earlier stages of callus induction and proliferation rather than medium composition at the late stages of suspension maintenance. These results suggest that suspension cell cultures of Vietnamese ginseng have a high potential for the biotechnological production of biomass containing ginsenosides, particularly of oleanolic acid and ocotillol groups, and may become a sustainable source of raw materials for functional foods, cosmetics, and natural health products. Further studies should explore bioreactor cultivation of the most perspective cell lines (PV-70 and PV-70-SH) and assessment of the biological activity of the resulting cell biomass. The specific spectra of ginsenosides detected in the cell lines may also provoke further investigation.

## Data availability statement

The data presented in the study are deposited in the MassIVE repository, accession number MSV00009359ucsd.e https://massive.ucsd.edu/ProteoSAFe/dataset.jsp?task=b9fd46ced77b4e86ab8cc4004285bc15.

## Author contributions

MT: Conceptualization, Data curation, Funding acquisition, Investigation, Methodology, Project administration, Resources, Supervision, Visualization, Writing – original draft, Writing – review & editing. ML: Data curation, Formal analysis, Investigation, Methodology, Software, Validation, Visualization, Writing – original draft, Writing – review & editing. TT: Data curation, Investigation, Methodology, Software, Validation, Writing – original draft, Writing – review & editing. OP: Investigation, Methodology, Validation, Writing – review & editing. EP: Data curation, Funding acquisition, Project administration, Resources, Supervision, Validation, Visualization, Writing – original draft, Writing – review & editing. OK: Data curation, Formal analysis, Investigation, Methodology, Resources, Supervision, Validation, Writing – review & editing. PM: Investigation, Methodology, Resources, Software, Writing – review & editing. YI: Data curation, Formal Analysis, Investigation, Methodology, Resources, Software, Supervision, Validation, Visualization, Writing – review & editing. EV: Data curation, Investigation, Methodology, Software, Writing – review & editing. IR: Conceptualization, Data curation, Methodology, Project administration, Resources, Software, Supervision, Validation, Writing – review & editing. AN: Conceptualization, Data curation, Funding acquisition, Project administration, Resources, Supervision, Validation, Writing – review & editing.
