# Supplementary material for: Suspension cell cultures of Panax vietnamensis as a biotechnological source of ginsenosides: growth, cytology, and ginsenoside profile assessment
Source: Front Plant Sci. 2024 Feb 26;15:1349494. doi: 10.3389/fpls.2024.1349494 (PMC10926444; doi:10.3389/fpls.2024.1349494)
Supplement: Supplementary file 1 [file DataSheet_1.docx]

Supplementary Materials

**Table S1.** Growth phases of *P. vietnamensis* suspension cell cultures (determined based on dry weight from the growth curves plotted in the semi-logarithmic coordinates shown in Fig. S3)

| **Suspension cell line** | **Growth cycle phase (days after inoculation)*** | | | | | |
| --- | --- | --- | --- | --- | --- | --- |
|  | **Lag phase** | **Growth acceleration**  **phase** | **Exponential**  **phase** | **Growth retardation phase** | **Stationary phase** | **Degradation phase** |
| ***Inoculum density X_0_* = 0.5 gDW L^-1^** | | | | | | |
| **PV-70** | 0-9 | 9-18 | 18-37 | 37> | n\o | n\o |
| **PV-4** | 0-2 | 2-7 | 7-16 | 16-32 | 32 > | n\o |
| **PV-71** | 0-2 | 2-7 | 7-21 | 21-24 | 24-30 | 30 > |
| **PV-70-SH** | 0-2 | 2-7 | 7-31 | 30 > | n\o | n\o |
| **PV-4-SH** | 0-2 | 2-7 | 7-28 | 28-32 | 32 > | n\o |
| **PV-71-SH** | 0-6 | 6-8 | 8-27 | 27-37 | 37 > | n\o |
| ***Inoculum density X_0_* = 1.0 gDW L^-1^** | | | | | | |
| **PV-70** | 0-2 | 2-8 | 8-21 | 21-35 | 21-35 | 35 > |
| **PV-4** | 0-4 | 4-7 | 7-18 | 18-28 | 28-32 | 32 > |
| **PV-71** | 0-3 | 3-6 | 6-18 | 18-32 | 32 > | n\o |
| **PV-70-SH** | 0-3 | 3-6 | 6-21 | 21-32 | 32 > | n\o |
| **PV-4-SH** | 0-6 | 6-8 | 8-24 | 24-31 | 31 > | n\o |
| **PV-71-SH** | 0-4 | 4-11 | 11-21 | 21-28 | 28-35 | 35 > |

* - Days are counted after inoculation to a fresh medium.

n\o - phase was not observed within the time frame of the experiment.

**Table S2.** Triterpene saponins annotated in the combined sample of six *P. vietnamensis* suspension cell culture lines by reversed-phase ultra-high-performance liquid chromatography—tandem mass spectrometry (UHPLC-Orbitrap-MS/MS) and ultra-performance liquid chromatography—mass spectrometry (UPLC-TOF-MS).

| Aglycon type | Compound Name (Abbreviation) | Molecular Formula | Ion Type | Calculated (*m/z*) | UHPLC-Orbitrap-MS/MS | | | | UPLC-TOF-MS | | | Reference |
| --- | --- | --- | --- | --- | --- | --- | --- | --- | --- | --- | --- | --- |
|  |  |  |  |  | *t_R_* (min) | Experimental (*m/z*) | Error (ppm) | Fragmentation pattern | *t_R_* (min) | Experimental (*m/z*) | Error (ppm) |  |
| OCT | Vinaginsenoside R1 (VinaR1) | C_44_H_74_O_15_ | [M-H+FA]^-^ | 887.5010 | 25.75 | 887.5018 | -0.9 | 841.4952, 799.4868, 653.4265, 635.4161, 491.3728 | 16.35 | 887.5516 | -57.0 | (Xia et al., 2022; Koo et al., 2023) |
| PPD | Malonyl ginsenoside Rb2/Rb3 (Mal-Rb2/Rb3) | C_56_H_92_O_25_ | [M-H]^-^ | 1163.5855 | 27.08 | 1163.5864 | -0.8 | 783.4905, 621.4371, 459.3834 | 25.63 | 1163.6382 | -45.3 | (Koo et al., 2023) |
| PPD | Malonyl ginsenoside Rb2/Rb3 isomer (Mal-Rb2/Rb3 isomer) | C_56_H_92_O_25_ | [M-H]^-^ | 1163.5855 | 27.18 | 1163.5866 | -0.9 | 783.4897, 621.4377, 459.3855 | 25.75 | 1163.6382 | -45.3 | (Koo et al., 2023) |
| PPD | Ginsenoside Rb1* (Rb1) | C_54_H_92_O_23_ | [M-H]^-^ | 1107.5957 | 26.62 | 1107.5976 | -1.7 | 1107.5967, 945.5418, 783.4936, 621.4374 | 24.82 | 1107.7198 | -112.0 | (Xia et al., 2022; Koo et al., 2023) |
|  |  |  | [M-H+FA]^-^ | 1153.6011 |  | 1153.6021 | -0.9 | 783.4905, 621.4374 |  | 1153.6633 | -53.9 |  |
| PPD | Malonyl ginsenoside Rb1 (Mal-Rb1) | C_57_H_94_O_26_ | [M-H]^-^ | 1193.5960 | 26.73 | 1193.5975 | -1.3 | — | 25.01 | 1193.6533 | -48.0 | (Koo et al., 2023) |
| PPD | Malonyl ginsenoside Rb1 isomer (Mal-Rb1 isomer) | C_57_H_94_O_26_ | [M-H]^-^ | 1193.5960 | 26.83 | 1193.5970 | -0.8 | 1149.6063, 945.5395, 783.4880, 459.3864 | 25.09 | 1193.6533 | -48.0 | (Koo et al., 2023) |
| OA | Chikusetsusaponin IV/isomer (1) (CS-IV/isomer (1)) | C_47_H_74_O_18_ | [M-H]^-^ | 925.4803 | 27.19 | 925.4817 | -1.5 | 925.4814, 775.4249, 613.3762, 569.3853 | 25.47 | 925.5294 | -53.1 | (Jinbiao et al., 2022; Koo et al., 2023) |
| OA | Chikusetsusaponin IV/isomer (2) (CS-IV/isomer (2)) | C_47_H_74_O_18_ | [M-H]^-^ | 925.4803 | 27.25 | 925.4818 | -1.6 | 925.4813, 793.4344, 613.3734, 569.3857 | 25.59 | 925.5294 | -53.1 | (Jinbiao et al., 2022; Koo et al., 2023) |
| OA | Chikusetsusaponin IV/isomer (3) (CS-IV/isomer (3)) | C_47_H_74_O_18_ | [M-H]^-^ | 925.4803 | 27.38 | 925.4822 | -2.1 | 925.4820, 763.4291, 701.4257, 551.3762 | 25.85 | 925.5294 | -53.1 | (Jinbiao et al., 2022; Koo et al., 2023) |
| OA | Chikusetsusaponin IV/isomer (4) (CS-IV/isomer (4)) | C_47_H_74_O_18_ | [M-H]^-^ | 925.4803 | 28.46 | 925.4805 | -0.2 | 925.4808, 793.4381, 775.4225, 763.4274, 745.4175, 731.4388, 701.4251, 613.3743, 569.3856, 551.3750, 455.3538 | 27.05 | 925.5294 | -53.1 | (Jinbiao et al., 2022; Koo et al., 2023) |
| OA | Chikusetsusaponin IV/isomer (5) (CS-IV/isomer (5)) | C_47_H_74_O_18_ | [M-H]^-^ | 925.4803 | 28.58 | 925.4808 | -0.5 | 925.4810, 793.4424, 731.4381, 569.3853, 551.3749, 455.3534 |  |  |  | (Jinbiao et al., 2022; Koo et al., 2023) |
| OA | Ginsenoside R0* (Chikusetsusaponin V, R0) | C_48_H_76_O_19_ | [M-H]^-^ | 955.4908 | 26.98 | 955.4908 | 0.0 | 955.4921, 793.4393, 731.4370, 569.3854 | 25.18 | 955.5534 | -65.6 | (Xia et al., 2022; Koo et al., 2023) |
| OA | Ginsenoside R0 isomer  (Chikusetsusaponin V isomer, R0 isomer) | C_48_H_76_O_19_ | [M-H]^-^ | 955.4908 | 27.09 | 955.4915 | -0.7 | 955.4911, 793.4388, 731.4376, 569.3853, 551.3748, 455.3539 | 25.35 | 955.5534 | -65.6 | (Koo et al., 2023) |

* - ginsenosides annotated using commercial standards. PPD – protopanaxadiol group; OCT – ocotillol group; OA – oleanolic acid group.

**Table S3**. Peak areas of ginsenosides in the *P. vietnamensis* suspension cell cultures, lines PV-4-SH, PV-71-SH, PV-70-SH, PV-4, PV-71, and PV-70, analyzed by UPLC-TOF-MS.

| **Ginsenoside/Cell line** | **PV-4-SH** | **PV-71** | **PV-71-SH** | **PV-4** | **PV-70** | **PV-70-SH** |
| --- | --- | --- | --- | --- | --- | --- |
| **Rb1** | − | 174±18 | 108±33 | 6446±1156 | 74546±8485 | 67060±3662 |
| **Mal-Rb1** | − | − | − | 652±168 | 15565±1697 | 28053±2085 |
| **Mal-Rb1 isomer** | − | − | 186±61 | 2650±822 | 53236±6123 | 41728±11456 |
| **Mal-Rb2/Rb3** | 28±10 | 621±155 | 40428±7273 | 285±30 | 61935±4428 | 54513±2984 |
| **Mal-Rb2/Rb3 isomer** | 265±67 | 296±86 | 17938±3873 | 983±131 | 130262±5861 | 84432±4305 |
| **VinaR1** | − | − | − | 153±55 | 5699±1357 | 290234±28442 |
| **R0** | 4604±1086 | 22392±542 | 2564±408 | 183924±24117 | 19708±436 | 51506±2241 |
| **R0 isomer** | 26730±2011 | 16876±754 | 10612±457 | 21173±1619 | 25989±1253 | 24625±3772 |
| **CS-IV/isomer (1)** | 2903±329 | 537±28 | 1263±121 | 26660±3678 | 16400±1743 | 8640±316 |
| **CS-IV/isomer (2)** | 665±94 | 722±29 | 512±62 | 19952±4914 | 4342±1177 | 10975±508 |
| **CS-IV/isomer (3)** | 8030±565 | 766±133 | 461±36 | 4308±570 | 4449±1449 | 9288±1660 |
| **CS-IV/isomer (4+5)** | 47812±7823 | 26469±2253 | 31782±3189 | 141208±10794 | 83884±5986 | 88000±14474 |

“−” – compound not found


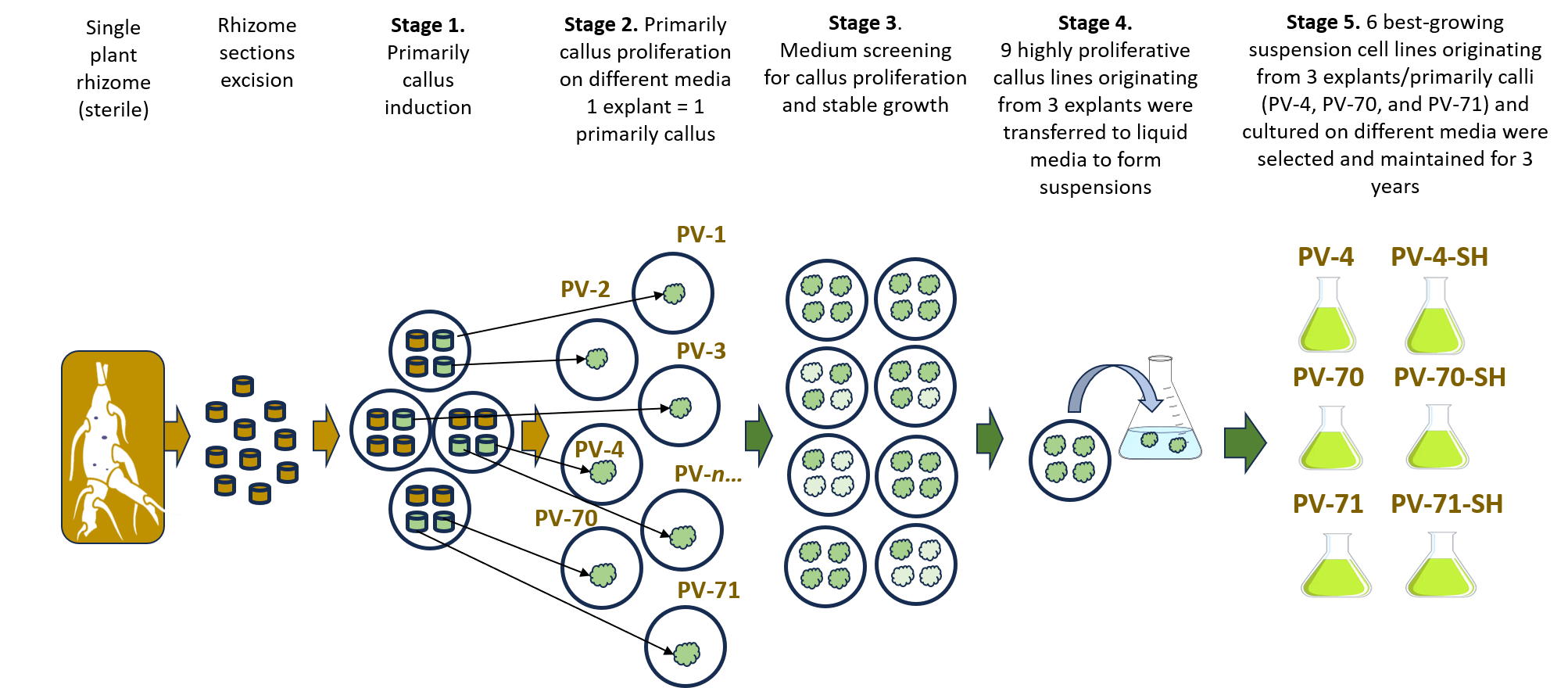


**Figure S1.** The scheme of a multi-stage process of callus and suspension culture induction from a single *Panax vietnamensis* rhizome, modified from (Sobolkova et al., 2018).

**Stage 1:** primarily callus was induced from different segments of a single rhizome on three different media with MS or B5 mineral salt base and different vitamin combination without growth regulators. **Stage 2:** primarily calli formed at the previous stage were transferred to proliferation media with MS mineral salt base and vitamins, sucrose or glucose as carbohydrate sources and different combinations of growth regulators (2,4-D, NAA, BA, and kinetin). Over 70 primarily callus lines were induced. The progeny of different explants were named PV-1, PV-2, etc.

**Stage 3:** the best-growing callus lines from the previous stage were cross-cultured to ten different media composed of MS or B5 mineral salt base, MS vitamins, 30 g L^-1^ sucrose and different combinations of growth regulators (2,4-D, NAA, BA, and kinetin), calli with low viability and slow growth were discarded.

At Stages 1-3, calli were maintained for 3 months, with monthly subcultures to fresh medium.

**Stage 4:** nine highly-proliferative callus lines originating from three initial explants, PV-4, PV-70, and PV-71, and cultured on different media were placed in liquid medium of the same composition in flasks to initiate suspensions. Suspensions were developed during 5 months with monthly subcultures to fresh media.

**Stage 5:** six best-growing suspension cell cultures were selected and maintained for 3 years on their respecitive media (Table 1 in the main text).

| **PV-70** |
| --- |
| 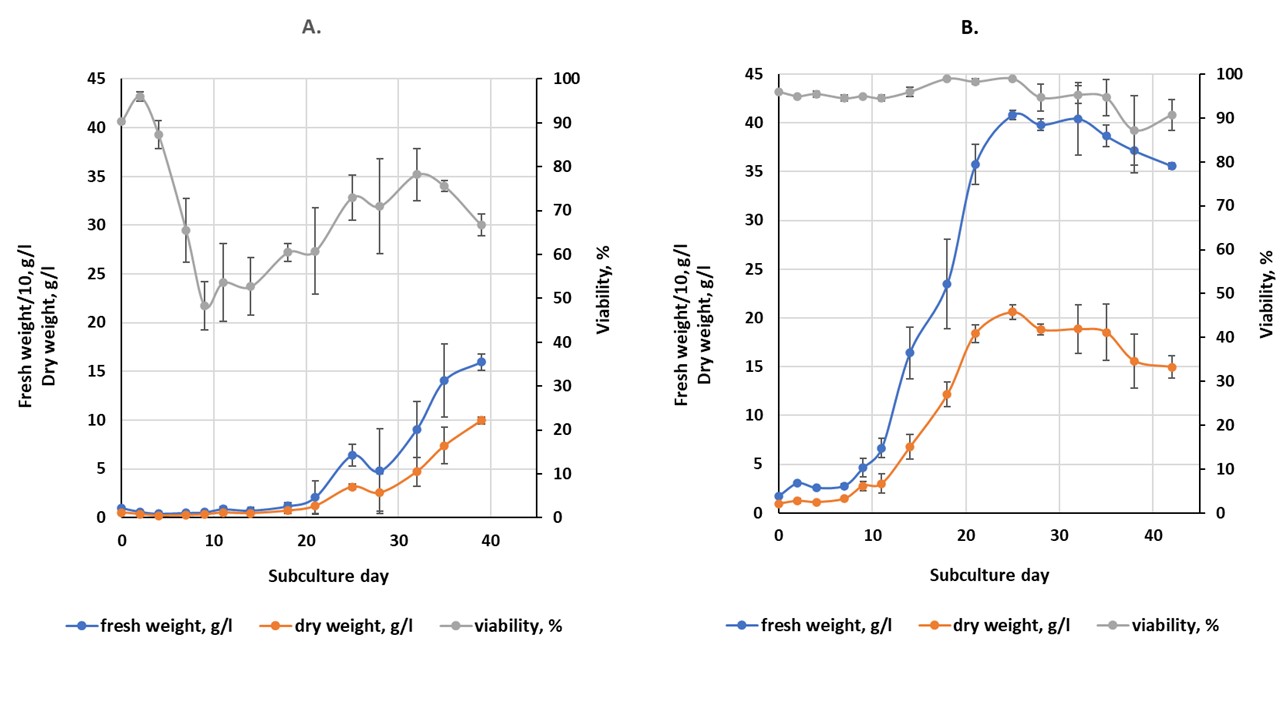 |
| **PV-71** |
| 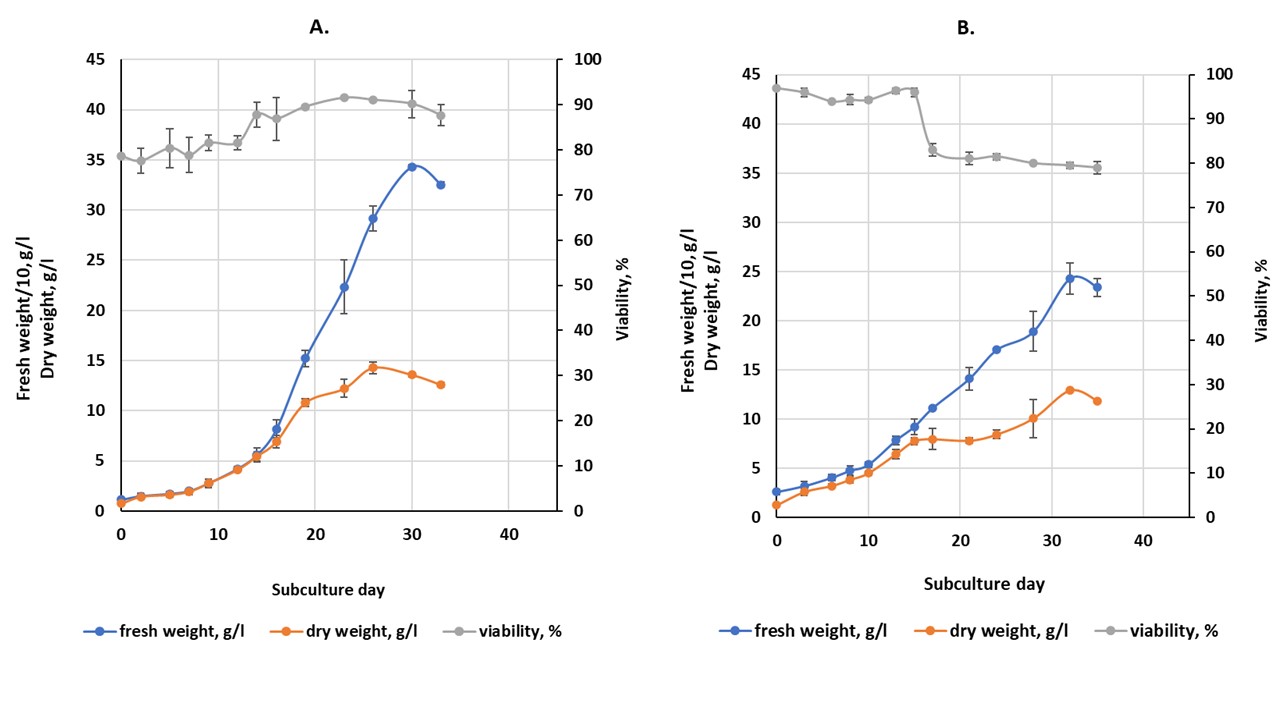 |
| **PV-4** |
| 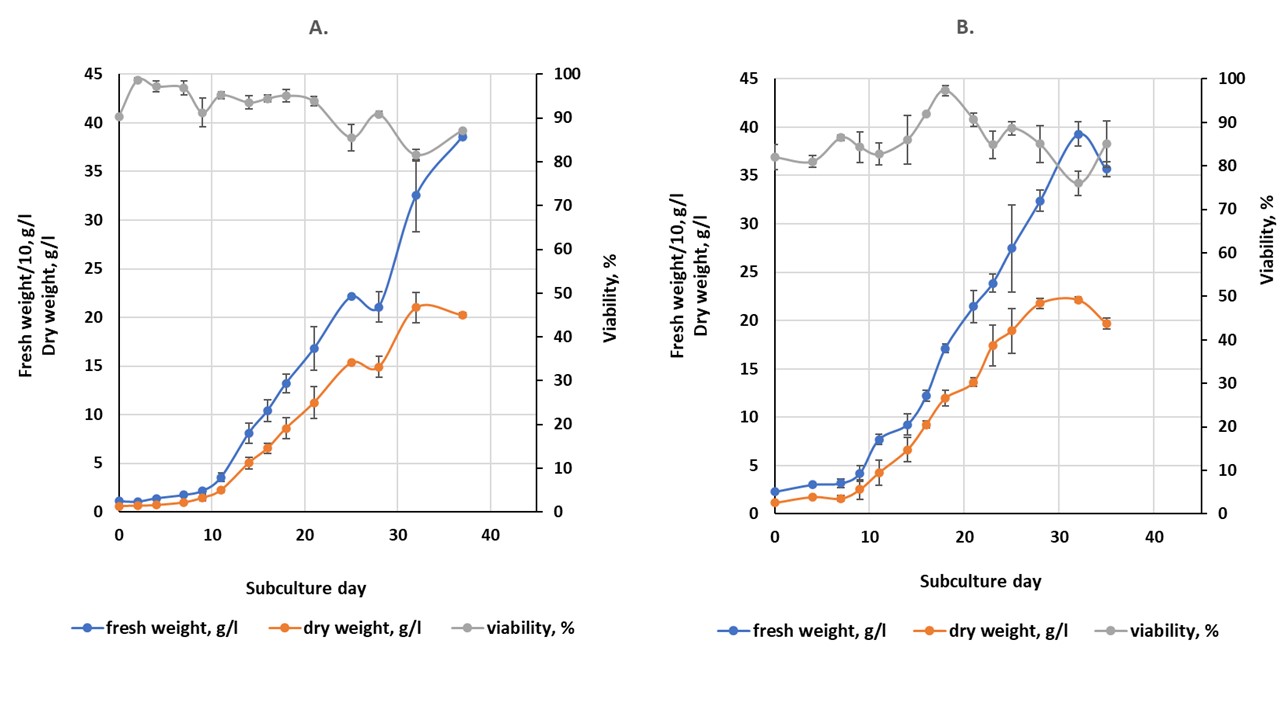 |
| **PV-70-SH** |
| 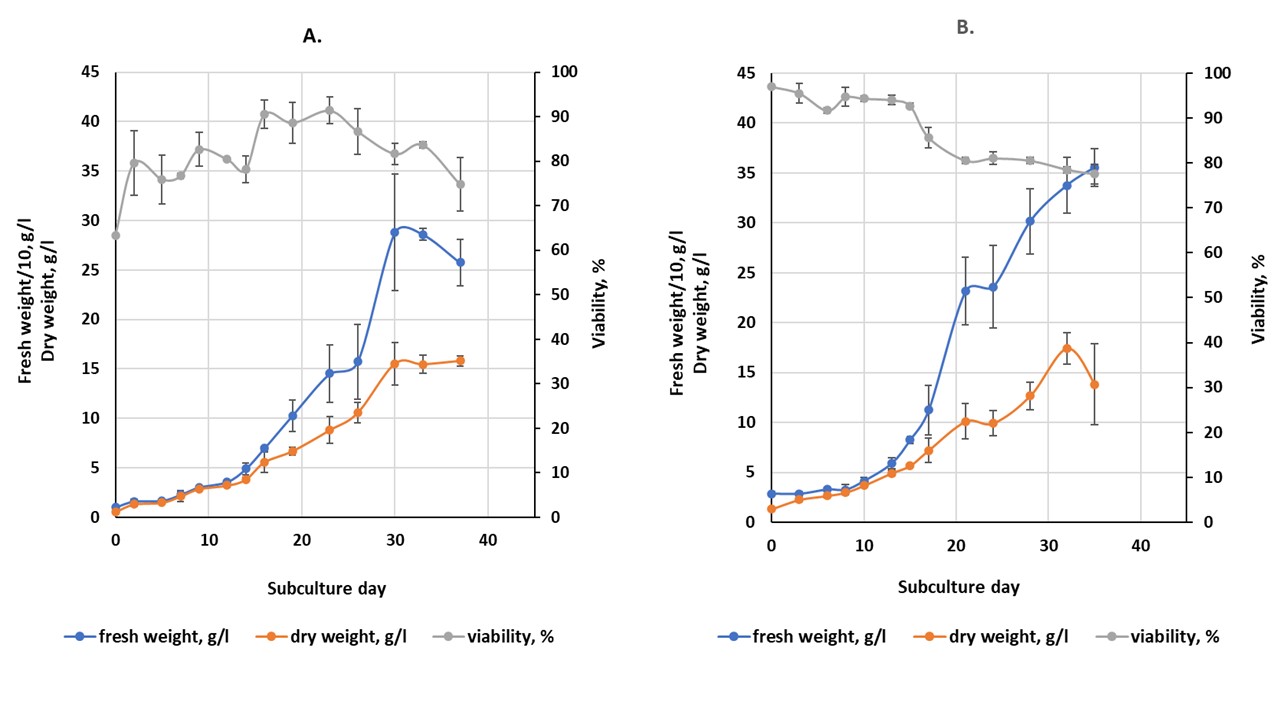 |
| **PV-71-SH** |
| 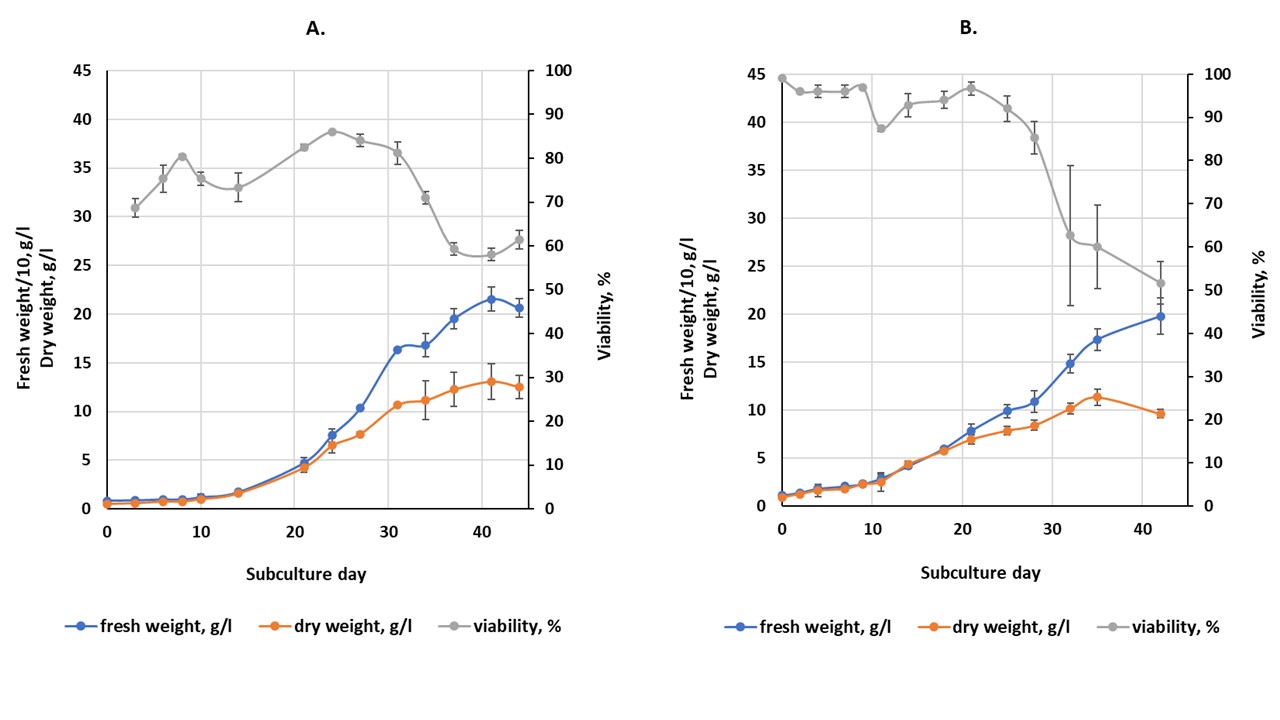 |
| **PV-4-SH** |
| 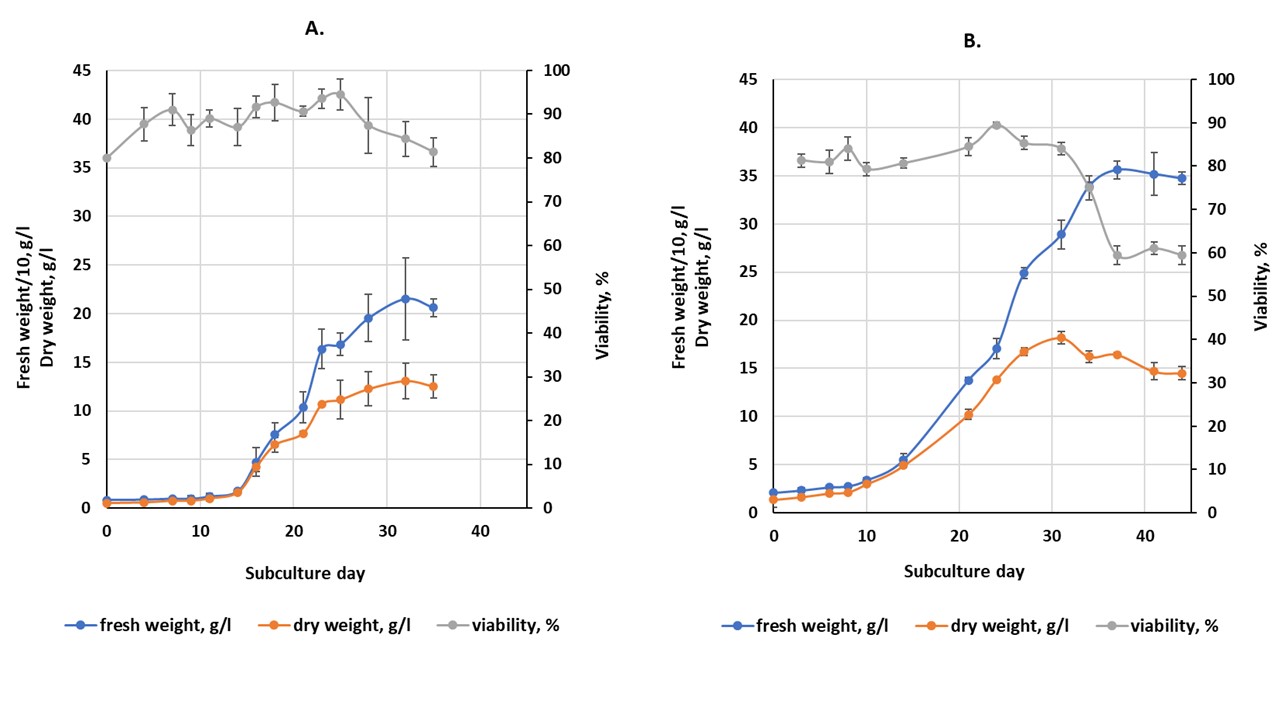 |

**Figure S2.** Growth curves of the suspension cell culture of *P. vietnamensis,* lines PV-70, PV-71, PV-4, PV-70-SH, PV-71-SH, and PV-4-SH, during cultivation in 250 mL flasks: fresh and dry weights and cell viability plotted in normal coordinates. **(A)** inoculum density *X_0_* = 0.5 gDW L^-1^; **(B)**  inoculum density *X_0_* = 1.0 gDW L^-1^. For every cell line, data are presented as mean values from three flasks (*n* = 3) and standard deviations for each data point.

| A. | 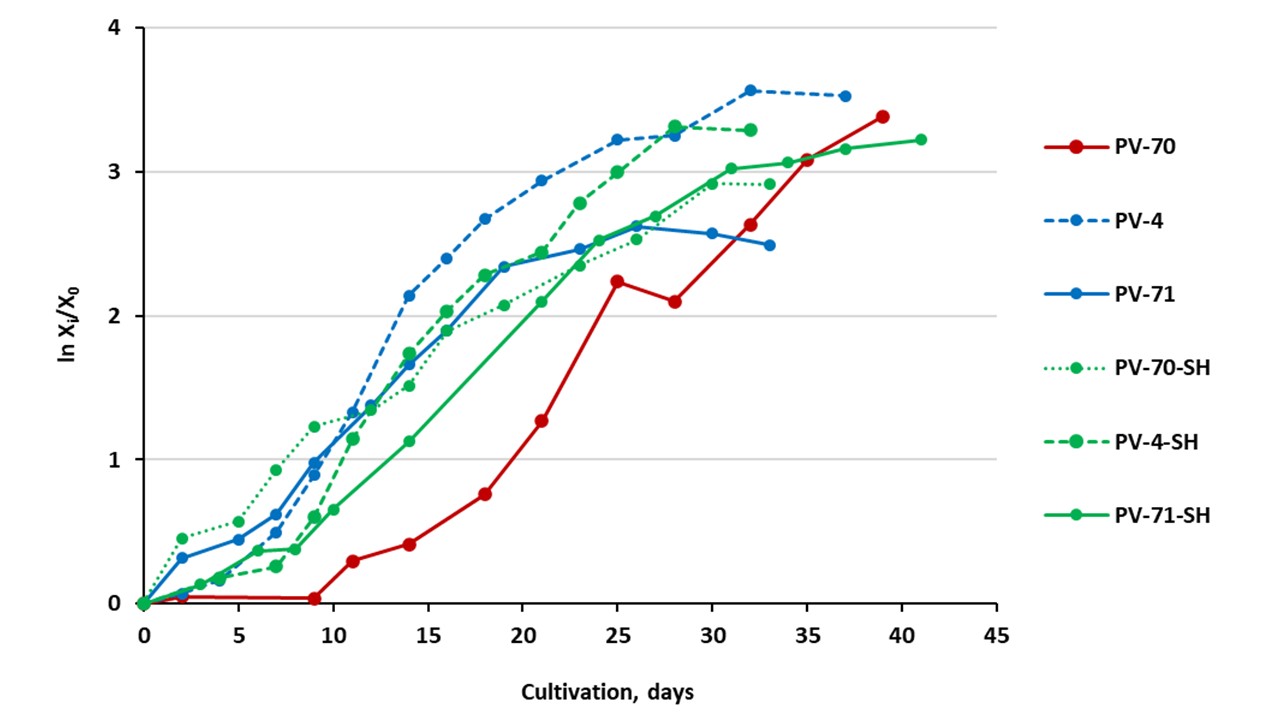 |
| --- | --- |
| B. | 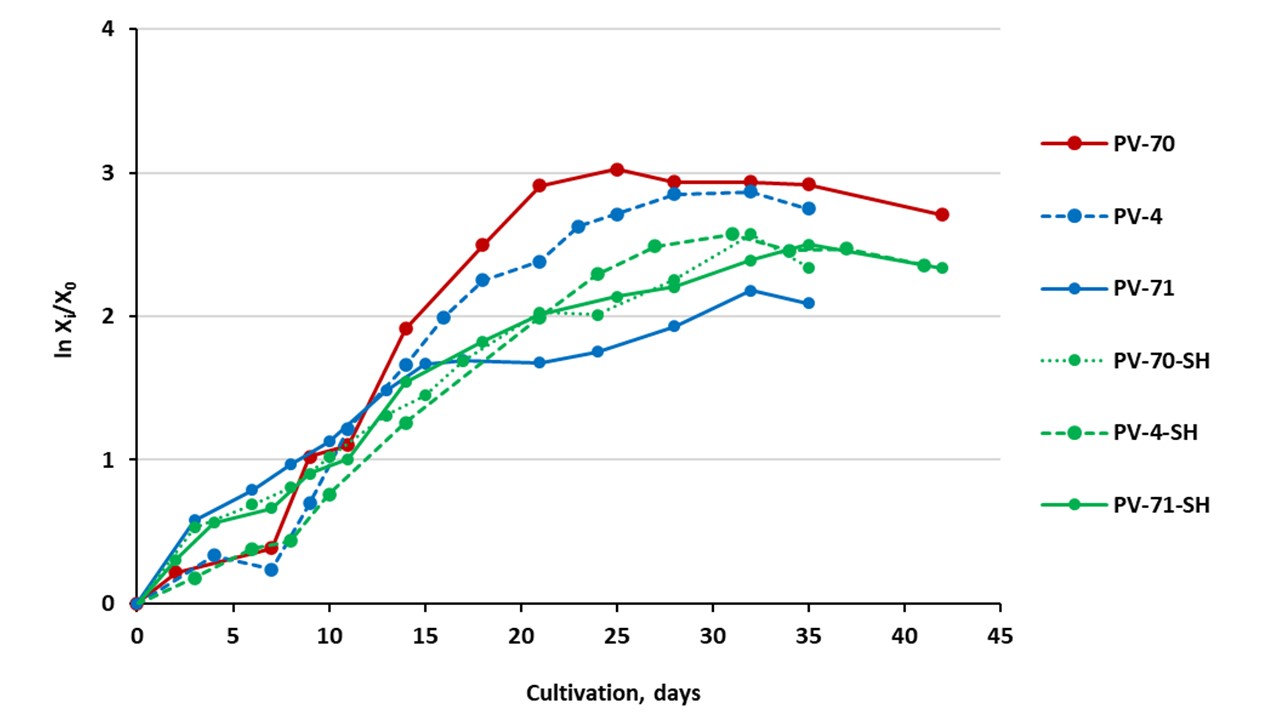 |

**Figure S3.** Growth curves (dry weight) of the suspension cell culture of *P. vietnamensis,* cell lines PV-70, PV-71, PV-4, PV-70-SH, PV-71-SH, and PV-4-SH, during cultivation in 250 mL flasks with the inoculum density *X_0_* =0.5 gDW L^-1^ **(A)** and *X_0_* =1.0 gDW L^-1^ **(B)** plotted in semi-logarithmic coordinates.


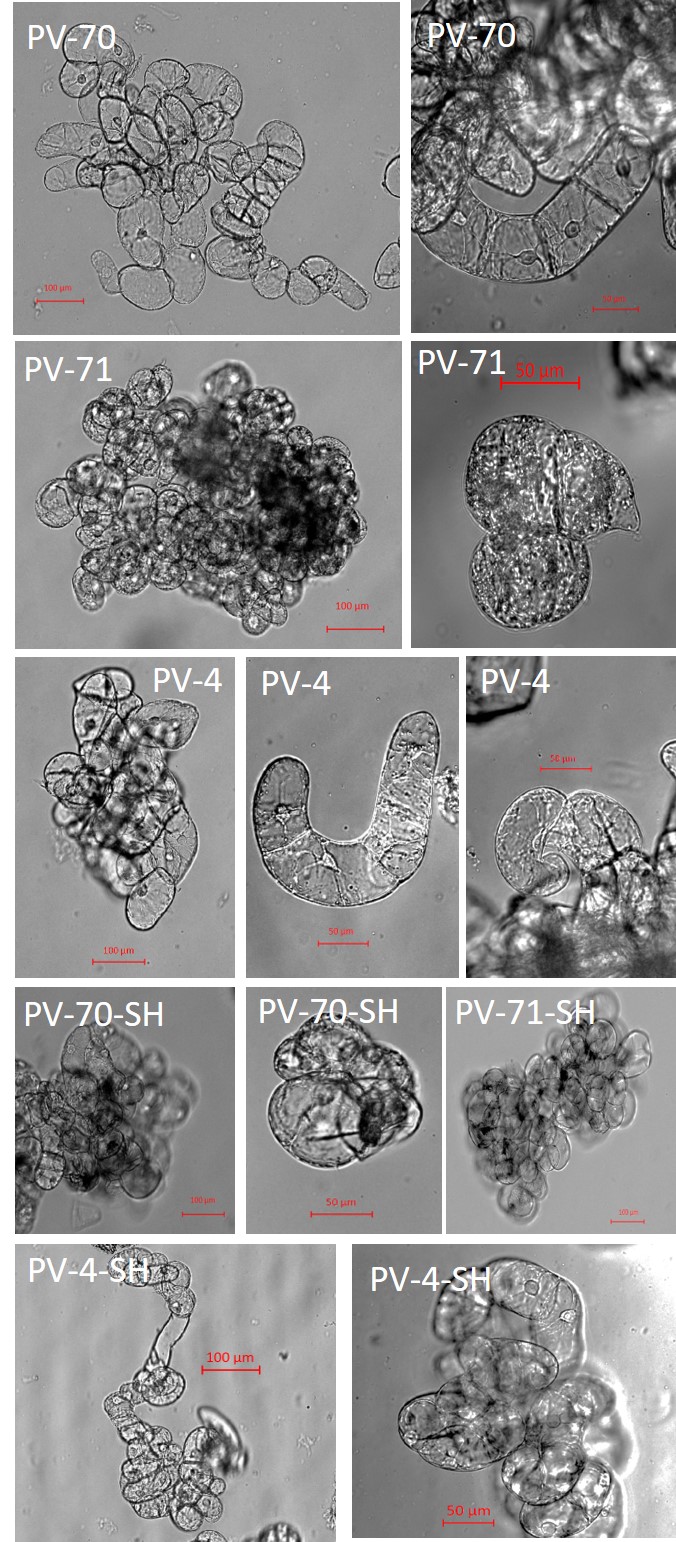


**Figure S4.** Photographs of cells and cell aggregates of different *P. vietnamensis* cell lines at the exponential growth phase.


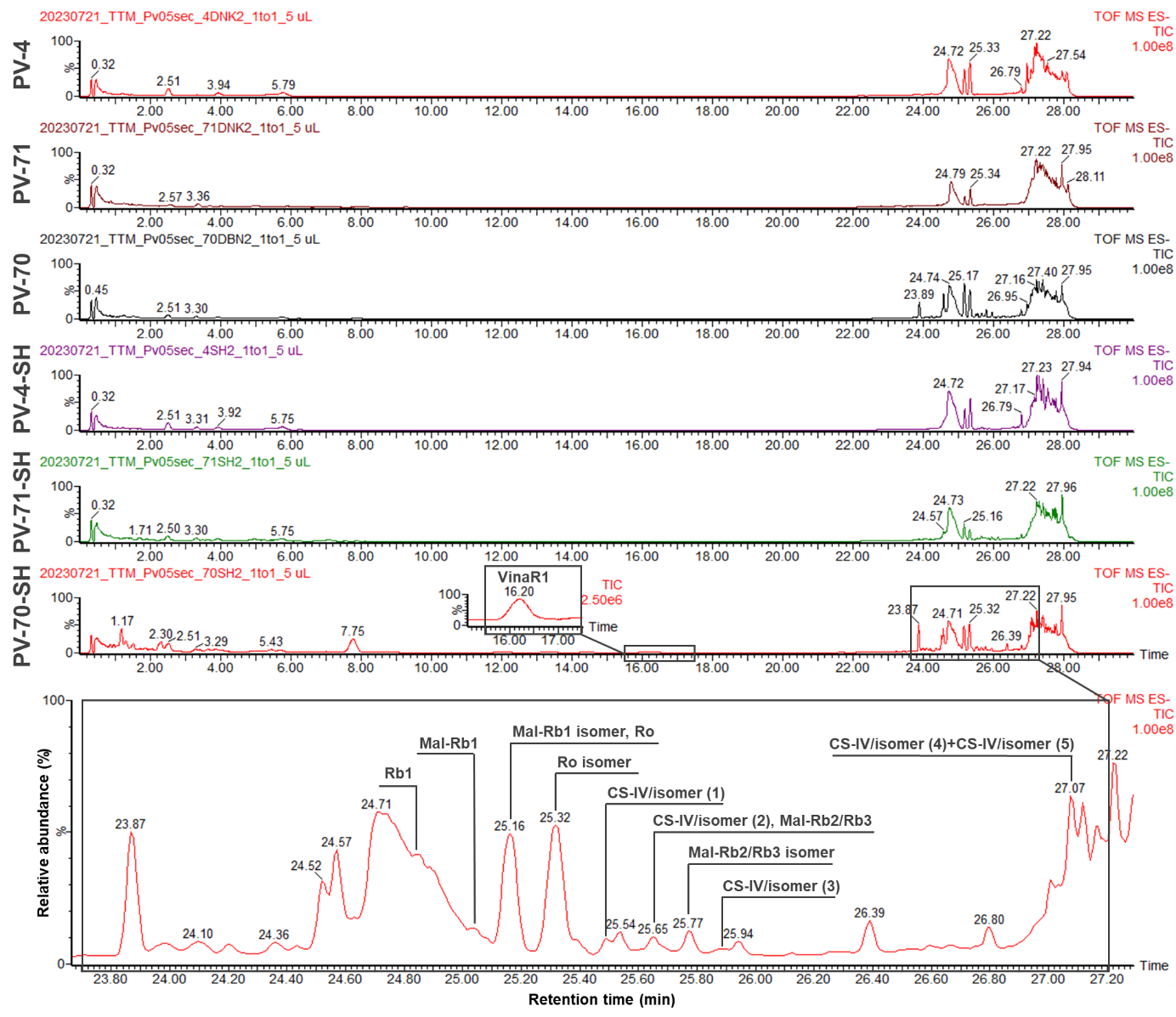


**Figure S5.** UPLC-TOF-MS - total ion current (TIC) chromatograms of methanolic extracts of six lines of *P. vietnamensis* suspension cell culture.


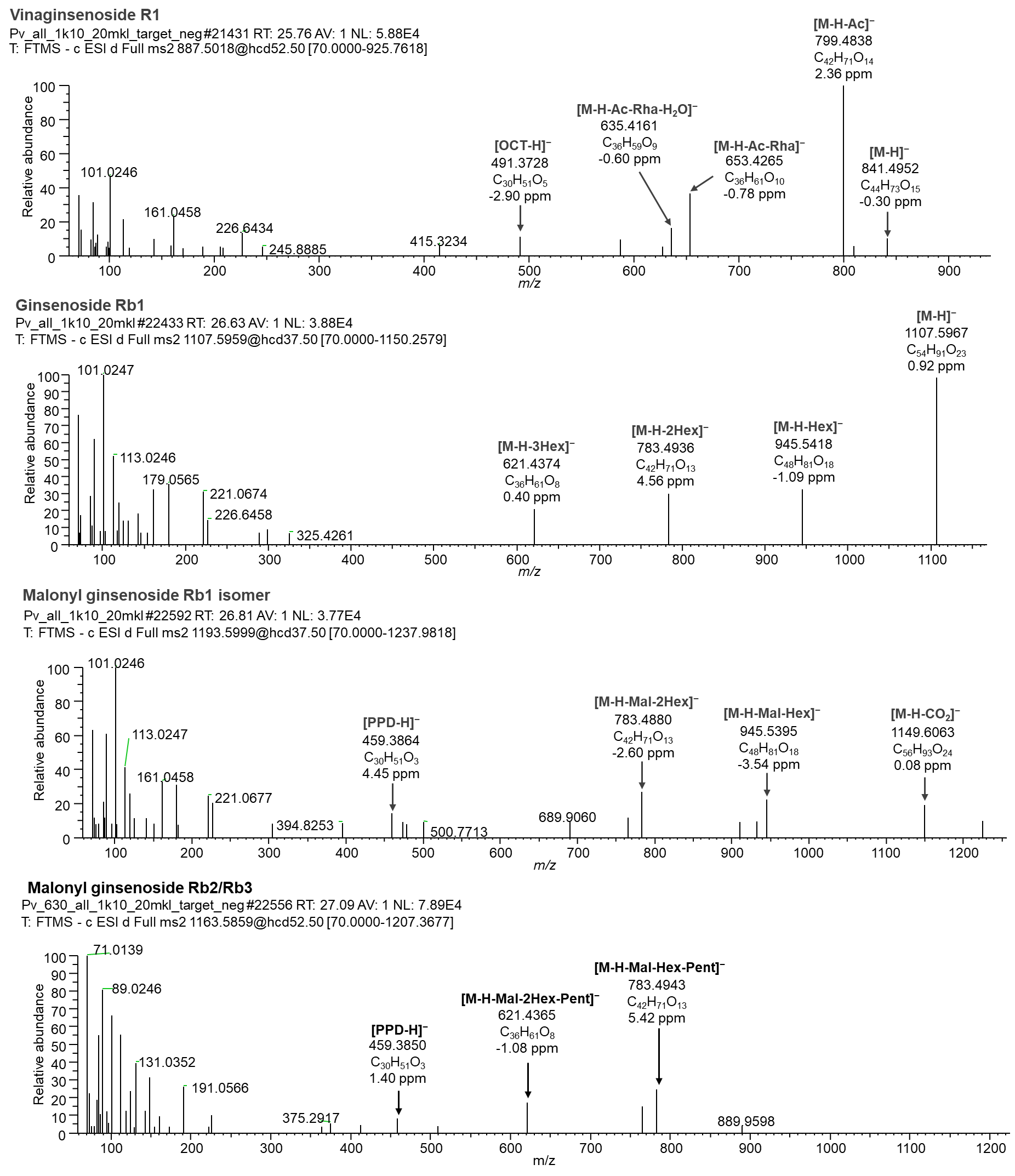


**Figure S6.** Fragmentation spectra of *m/z* 887.5 at t_R_ 25.8 min corresponding to Vinaginsenoside R1, *m/z* 1107.6 at t_R_ 26.6 min corresponding to Ginsenoside Rb1 and *m/z* 1193.6 at t_R_ 26.8 min corresponding to Malonyl ginsenoside Rb1 isomer annotated in the combined sample out of all six lines of *P. vietnamensis* suspension cell cultures. Abbreviations: Ac – acetyl (C_2_H_2_O), Rha – rhamnose (C_6_H_10_O_4_), Hex – hexose (C_6_H_10_O_5_), Pent – pentose (C_5_H_8_O_4_), Mal – malonyl (C_3_H_2_O_3_).


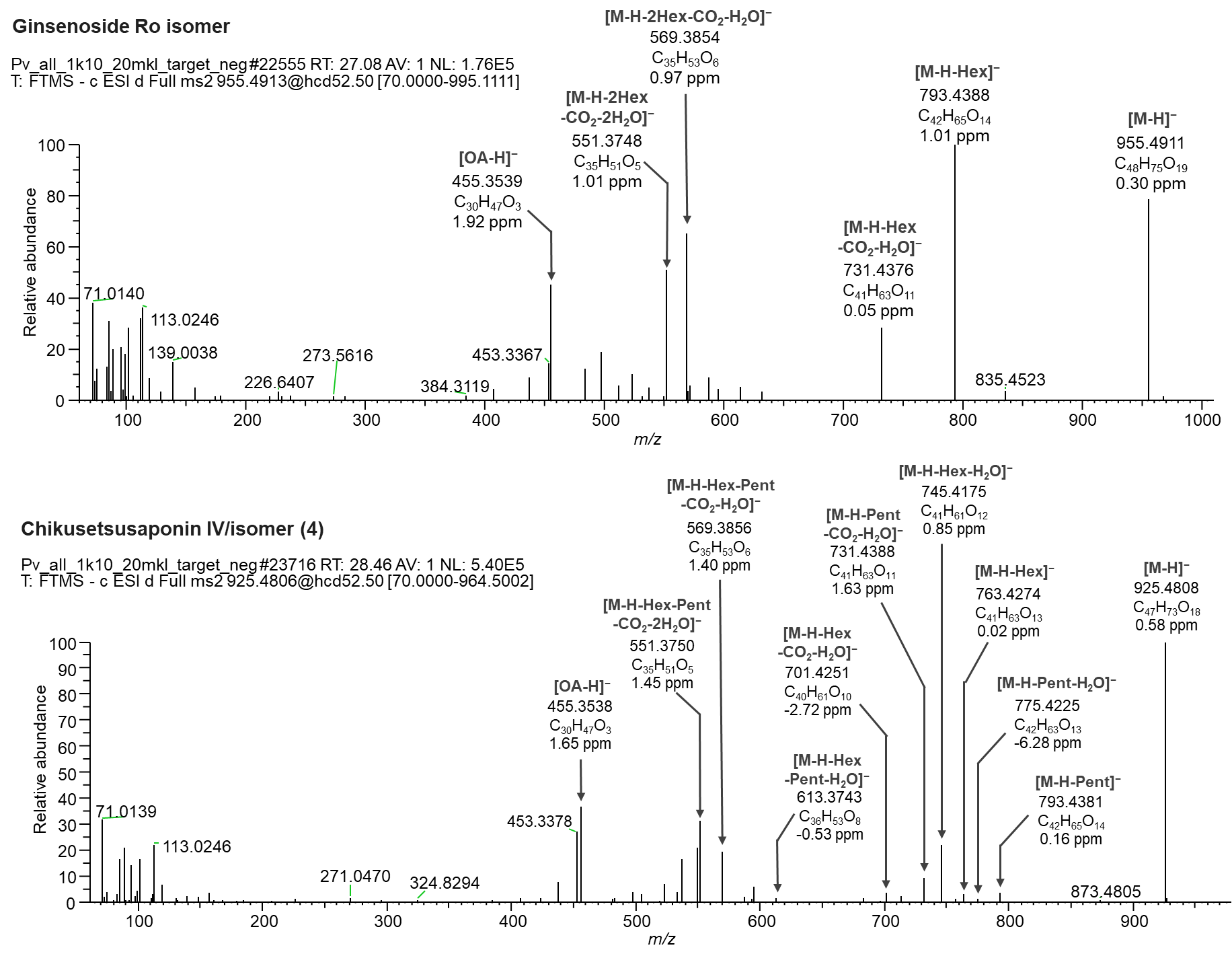


**Figure S7**. Fragmentation spectra of *m/z* 955.5 at t_R_ 27.1 min corresponding to Ginsenoside R0 isomer and *m/z* 925.5 at t_R_ 28.5 min corresponding to Chikusetsusaponin IV/isomer (4) annotated in the combined sample out of all six cell lines of *P. vietnamensis* suspension cell cultures. Abbreviations: Hex – hexose (C_6_H_10_O_5_), Pent – pentose (C_5_H_8_O_4_).
